# Additive Dose Response Models: Defining Synergy

**DOI:** 10.3389/fphar.2019.01384

**Published:** 2019-11-26

**Authors:** Simone Lederer, Tjeerd M. H. Dijkstra, Tom Heskes

**Affiliations:** ^1^Data Science, Institute for Computing and Information Sciences, Radbound University, Nijmegen, Netherlands; ^2^Max Planck Institute for Developmental Biology, Tübingen, Germany; ^3^Center for Integrative Neuroscience, University Tübingen, Tübingen, Germany

**Keywords:** synergy, Loewe Additivity, Bliss Independence, dose equivalence, General Isobole Equation, Explicit Mean Equation, Hill curve, lack-of-fit

## Abstract

In synergy studies, one focuses on compound combinations that promise a synergistic or antagonistic effect. With the help of high-throughput techniques, a huge amount of compound combinations can be screened and filtered for suitable candidates for a more detailed analysis. Those promising candidates are chosen based on the deviance between a measured response and an expected non-interactive response. A non-interactive response is based on a principle of no interaction, such as Loewe Additivity or Bliss Independence. In a previous study, we introduced, an explicit formulation of the hitherto implicitly defined Loewe Additivity, the so-called Explicit Mean Equation. In the current study we show that this Explicit Mean Equation outperforms the original implicit formulation of Loewe Additivity and Bliss Independence when measuring synergy in terms of the deviance between measured and expected response, called the lack-of-fit. Further, we show that computing synergy as lack-of-fit outperforms a parametric approach. We show this on two datasets of compound combinations that are categorized into synergistic, non-interactive, and antagonistic.

## Introduction

When combining a substance with other substances, one is generally interested in interaction effects. Those interaction effects are usually described as synergistic or antagonistic, dependent on whether the interaction is positive, resulting in greater effects than expected, or negative, resulting in smaller effects than expected. From data generated with high-throughput techniques, one is confronted with massive compound interaction screens. From those screens, one needs to filter for interesting candidates that exhibit an interaction effect. To quickly scan all interactions, a simple measure is needed. Based on that preprocessing scan, those filtered combination candidates can then be examined in greater detail. In such a quick scan, one focuses uniquely on the measured response and not on possible mechanisms of action of each compound.

To determine whether a combination of substances exhibits an interaction effect, it is crucial to determine a non-interactive effect. Only when deviance from that so-called null reference is observed, can one speak of an interactive effect (Lederer et al., 2018b). Over the last century, many principles of non-interaction have been introduced. For an extensive overview, refer to Greco et al. (1995) and Geary (2012). Two main principles for non-interactivity have survived the critics: Loewe Additivity (Loewe, 1928) and Bliss Independence (Bliss, 1939). The popularity of Loewe Additivity is based on its principle of sham combination which assumes no interaction when a compound is combined with itself. Other null reference models do not hold that assumption. An alternative is Bliss Independence, which assumes (statistical) independence between the combined compounds.

Independent of the indecisive opinions about the null reference, there are multiple proposals regarding how synergy can be measured given a null reference model. Many models are based on the concept of isoboles (Chou and Talalay, 1984; Gennings and Carter, 1995; Dawson et al., 2000 Minto et al., 2000). An isobole is the set of all dose combinations of the compounds that reach the same fixed effect, such as 50% of the maximal effect. Some suggest to measure synergy as the difference between an observed isobole and a reference isobole calculated from a null-reference model. Another way to quantify synergy on the basis of the isobole is to look at the curvature and arc-length of the longest isobole spanned over the measured response (Cokol et al., 2011). The general problem with the isobole approaches lies in the use of isoboles at fixed effect or dose ratios. The deviations measured from the isoboles (and hence synergy) are therefore only measured locally for that fixed effects or dose ratios. In order to measure all interaction effects, this method has to be applied to many fixed effects or dose ratios.

In this paper we measure synergy as the deviation over the entire response surface. One way to do so is the Combenefit method by measuring synergy in terms of volume between the expected and measured effect (Di Veroli et al., 2016). We will refer to it as a lack-of-fit method as it quantifies the lack of fit from the measured data to the null reference model. Another way of capturing the global variation is by introducing a synergy parameter α into the mathematical formulation of the response surface. This parameter α is fitted by minimizing the error between the measured effect and the α-dependent response surface. Such statistical definition of synergy allows for statistical testing of significance of the synergy parameter. Fitting a synergy parameter to the data as in the parametric approaches tends to be computationally more complex than computing the difference between the raw data and the null model as in the lack-of-fit approaches.

There is an increase in theoretical approaches to synergy, such as the recently re-discovered Hand model (Hand, 2000; Sinzger et al., 2019), which is a formulation of Loewe Additivity in form of a differential expression, or new ways of defining and measuring synergy, such as the ZIP model (Yadav et al., 2015), SynergyFinder (He and Tang, 2016), MuSyC (Meyer et al., 2019), and the copula model (Lambert and Dawson, 2019). It would be a large effort to compare these recent approaches with ours. An extensive comparison of the models has recently been made in Meyer et al. (2019). Hence we focus on the two main principles, Loewe Additivity and Bliss Independence.

As the research area of synergy evolved from different disciplines, different terminologies are in common use. While in pharmacology, one refers to the Loewe model, in toxicology, the same principle is called concentration addition. The response can be measured among others in growth rate, survival, or death. It is usually referred to as the measured or phenotype effect or as cell survival. In this study we interchange the terms response and effect.

When measuring a compound combination, one also measures each agent individually. The dose or concentration is typically some biological compound per unit of weight when using animal or plant models or per unit of volume when using a cell-based assay. However, it can also be an agent of a different type for example a dose of radiation as used in modern combination therapies for cancer (Nature Biomedical Engineering, 2018). This individual response is called mono-therapeutic response (Di Veroli et al., 2016) or single compound effect. We prefer a more statistical terminology and refer to it as conditional response or conditional effect. We speak of synergy when referring to a general interaction effect, be it synergistic or antagonistic. In the detailed analysis where the direction of interaction is of importance, we clearly differentiate between a synergistic or antagonistic effect. With **“record”** we refer to all measurements taken of one cell line or organism which is exposed to all combinations of two compounds. In other literature, this is referred to as response matrix (Lehar et al., 2007; Yadav et al., 2015).

In *Theory*, we give a short introduction to the two null response principles, Loewe Additivity and Bliss Independence. We explain in detail several null reference models that build on those principles. We introduce synergy as any effect different from an interaction free model in *Methods*. There, we also introduce the parametrized and lack-of-fit synergy approaches. In *Material*, we introduce two datasets that come with a categorization into synergistic, non-interactive, and antagonistic. We use these two datasets to compute a synergy score for each record per model and method, that we introduced in *Theory* and *Methods*. Based on the comparison of the computed and provided synergy scores, we evaluate the models and methods in *Results*.

## Materials and Methods

### Theory

Before one can decide whether a compound combination exhibits a synergistic effect, one needs to decide on the expected effect assuming no interaction between the compounds. Such so-called null reference models are constructed from the conditional (mono-therapeutic) dose–response curves of each of the compounds, which we denote by *f_j_* (*x_j_*) for every compound *j* ∈ {1,2}. Null reference models extend the conditional dose–response curves to a (null-reference) surface spanned between the two conditional responses. We denote the surface as *f* (*x*_1_, *x*_2_) such that

(1) $$f(x_{1},0)=f_{1}(x_{1})$$

and

(2) $$f(0,x_{2})=f_{2}(x_{2}).$$

Thus, the conditional response curves are the boundary conditions of the null reference surface. For this study, we focus on Hill curves to model the conditional dose–responses. More detailed information can be found in **Supplementary Material**.

#### Loewe Additivity

Loewe Additivity builds on the concepts of sham combination and dose equivalence. The first concept is the idea that a compound does not interact with itself. The latter concept assumes that both compounds that reach the same effect can be interchanged. Therefore, any linear combination of fractions of those doses which reach the effect individually and, summed up, are equal to one, yields that exact same effect. Mathematically speaking, if dose $x_{1}^{*}$ from the first compound reaches the same effect as dose $x_{2}^{*}$ from the second compound, then any dose combination (*x*_1_, *x*_2_), for which

(3) $$\frac{x_{1}}{x_{1}^{*}}+\frac{x_{2}}{x_{2}^{*}}=1$$

holds, should yield the same effect as $x_{2}^{*}$ and $x_{1}^{*}$. As this idea can be generalized to any effect *y*, one gets

(4) $$\frac{x_{1}}{f_{1}^{-1}(y)}+\frac{x_{2}}{f_{2}^{-1}(y)}=1,$$

where $x_{1}^{*}$ and $x_{2}^{*}$ are replaced with $f_{1}^{-1}(y)$ and $f_{2}^{-1}(y)$, the inverse functions of Hill curves, respectively. For a fixed effect *y*, Eq. 4 defines an isobole, which is in mathematical terms a contour line. Hence the name of this model: the General Isobole Equation. It is an implicit formulation as the effect *y* of a dose combination (*x*_1_, *x*_2_) is implicitly given in Eq. 4. In the following we use the mathematical notation for the General Isobole Equation *f*_GI_ (*x*_1_, *x*_2_) = *y* with *y* being the solution to Eq. 4.

It was shown by Lederer et al. (2018b) that the principle of Loewe Additivity is based on a so-called Loewe Additivity Consistency Condition (LACC). This condition is that it should not matter whether equivalent doses of two compounds are expressed in terms of the first or the second. Under the assumption of the LACC being valid, Lederer et al. (2018b) have shown, that a null reference model can be formulated explicitly, by expressing the doses of one compound in terms of the other compound:

(5) $$f_{2\to 1}(x_{1},x_{2})=f_{1}\left(x_{1}+f_{1}^{-1}(f_{2}(x_{2}))\right)$$

(6) $$f_{1\to 2}(x_{1},x_{2})=f_{2}\left(f_{2}^{-1}(f_{1}(x_{1}))+x_{2}\right),$$

where $f_{1}^{-1}(f_{2}(x_{2}))$ is the dose *x*_1_ of compound one to reach the same effect of compound two with dose *x*_2_ (see **Figure S1** in **Supplementary Material**). For a detailed explanation, refer to Lederer et al. (2018b). Summing up this dose equivalent of the first compound with the dose of the first compound allows for the computation of the expected effect of the compound combination. With the two formulations above, the effect *y* of the dose combination (*x*_1_, *x*_2_) is expressed as the effect of either one compound to reach that same effect. Under the LACC, all three models, Eq. 4, Eq. 5, and Eq. 6 are equivalent. It was further shown, that, in order for the LACC to hold, conditional dose–response curves must be proportional to each other, i.e. being parallel shifted on the *x*-axis in log-space. It has been commented by Geary (2012) and shown in (Lederer et al., 2018b), that this consistency condition is often violated. Geary (2012) himself comments that it cannot be determined whether a response that lies between the two surfaces *f*_2→1_ (*x*_1_, *x*_2_) and *f*_1→2_ (*x*_1_, *x*_2_) is synergistic or antagonistic and hence should be treated as non-interactive. We refer to the envelope spanned between the two explicit surfaces *f*_2→1_ (*x*_1_, *x*_2_) and *f*_1→2_ (*x*_1_, *x*_2_) in Eq. 5 and Eq. 6 as *f*_geary_. In contrast to that, in an effort to take advantage of the explicit formulation and to counteract the different behavior of Eq. 5 and Eq. 6 in case of a violated LACC, Lederer et al. (2018b) introduced the so-called Explicit Mean Equation as mean of the two explicit formulations of Eq. 5 and Eq. 6:

(7) $$f_{mean}(x_{1},x_{2})=1/2\left(f_{2\to 1}(x_{1},x_{2})+f_{1\to 2}(x_{1},x_{2})\right).$$

A more extensive overview of Loewe Additivity and definition of null reference models together with visualizations can be found in Lederer et al. (2018b).

#### Bliss Independence

Bliss Independence assumes independent sites of action of the two compounds and was introduced a decade later than Loewe Additivity in (Bliss, 1939). Note that the formulation of Bliss Independence depends on the measurement of the effect. The best known formulation of Bliss Independence is based on monotonically increasing responses for increasing doses:

(8) $$g_{\text{bliss}}(x_{1},x_{2})=g_{1}(x_{1})+g_{2}(x_{2})-g_{1}(x_{1})g_{2}(x_{2}),$$

where *g*_1_ (*x_i_*) *=* 1*− f_i_* (*x_i_*) is a conditional response curve with increasing effect for increasing doses. In case the effect is measured in percent, i.e. *y*∈[0, 100], the interaction term needs to be divided by 100 to ensure the right dimensionality of the term.

Here, we measure the effect in terms of cell survival or growth inhibition. Therefore the conditional response curves are monotonically decreasing for increasing concentrations or doses.

(9) $$f_{\text{bliss}}(x_{1},x_{2})=f_{1}(x_{1})f_{2}(x_{2}).$$

The records are normalized to the response at *x*_1_ = 0, *x*_2_ = 0, thus *f*_1_(0) = *f*_2_(0) = 1. To arrive from Eq. 8 to Eq. 9, one replaces any *g* by 1 − *f*. Chou and Talalay (1984) derive the Bliss Independence from a first order Michaelis–Menten kinetic system with mutually non-exclusive inhibitors.

While there are many mathematical variations to the general concept of Loewe Additivity (here we introduced five null-reference models based on this methodology), there is generally only one way to compute Bliss Independence.

### Methods

The six models introduced in the previous section are null reference models in that they predict a response surface in the absence of compound interaction. We capture synergy in a single parameter to facilitate the screening process. This is different from other approaches, such as Chou and Talalay (1977), who measure synergy as deviation from a null-reference isobole without summarizing the deviation in a single parameter. The single parameter value is typically referred to as synergy- or α-score (Berenbaum, 1977). As we investigate two methods to quantify synergy, we introduce two synergy parameters α and γ, which measure the extent of synergy and are calculated with different methods (more details below). Both synergy scores α and γ are parametrized such that α = 0 or γ = 0 denote absence of an interaction effect. In case α or γ take a value different from zero, we speak of a non-additive, or interactive effect. A compound combination is, dependent on the sign of synergy parameter, one of the three following:

(10) α,γ{>0 synergistic=0 additive or non-interactive<0 antagonistic

Here, we measure synergy in two different ways, namely in fitting parametrized models or computing the lack-of-fit. The first method fits null reference models that are extended with a synergy parameter α. For these parametrized models α is computed by minimizing the square deviation between the measured response and the response spanned by the α-dependent model. For the second method the difference between a null reference model and the data is computed. For this method, which we refer to as lack-of-fit, the synergy score γ is defined as the volume that is spanned between the null reference model and the measured response.

Just as the conditional responses form the boundary condition for the null-reference surface (Eq. 1, Eq. 2), we want the conditional responses to be the boundary condition for all values of α. Explicitly, assuming a synergy model dependent on α is denoted by *f* (*x*_1_, *x*_2_ǀ α), then

(11) $$\begin{array}{c} f(x_{1},0|\alpha )=f_{1}(x_{1})\\ f(0,x_{2}|\alpha )=f_{2}(x_{2}) \end{array}\}\forall \alpha ,$$

with *f_i_* denoting the conditional response of compound *i*. We refer to Eq. 11 as the Synergy Desideratum. As we will see below, not all synergy models fulfill this property.

#### Parametrized Synergy

We extend the six null reference models introduced in *Theory* in Eq. 4–Eq. 9, including the Geary model, to parametrized synergy models. The extension of the General Isobole Equation is the popular Combination Index introduced by Berenbaum (1977) and Chou and Talalay (1984):

(12) $$\frac{x_{1}}{f_{1}^{-1}(y)}+\frac{x_{2}}{f_{2}^{-1}(y)}=1-\alpha .$$

Berenbaum originally equated the left-hand side of Eq. 4 to the so-called Combination Index *I*. Depending on *I* smaller, larger, or equal to 1, synergy, antagonism or non-interaction is indicated. For consistency with the other synergy models, we set *I* = 1 − α such that α matches the outcomes as listed in Eq. 10. In *Results* we will refer to this implicit model as *f*_CI_ (*x*_1_*,x*_2_ǀ *α*), where α is the parameter that minimizes the squared error between measured data and Eq. 12.

Note that this model violates the Synergy Desideratum in Eq. 11 as α not zero leads to deviations from the conditional responses. Explicitly, *f*_CI_ (*x*_1_, 0ǀα) = *f*_1_((1 − α) *x*_1_) ≠ *f*_1_(*x*_1_). Although the Combination Index model violates the Synergy Desideratum, in practice it performs quite well and is in widespread use.

The explicit formulations in Eq. 5 and Eq. 6 are equivalent to the General Isobole Equation, *f*_GI_ (*x*_1_, *x*_2_), given in Eq. 4, under the LACC (Lederer et al., 2018b), but different if the conditional responses are not proportional. The two explicit equations are in fact an extension of the 'cooperative effect synergy' proposed by Geary (2012) for compounds with qualitatively similar effects. For these explicit formulations in Eq. 5 and Eq. 6 we propose a model that captures the interaction based on the explicit formulations:

(13) $$f_{2\to 1}(x_{1},x_{2}|\alpha )=f_{1}\left(x_{1}+(1+\alpha )f_{1}^{-1}(f_{2}(x_{2}))\right)$$

(14) $$f_{1\to 2}(x_{1},x_{2}|\alpha )=f_{2}\left((1+\alpha )f_{2}^{-1}(f_{1}(x_{1}))+x_{2}\right).$$

With this, we can extend the Explicit Mean Equation model *f*_mean_ (*x*_1_, *x*_2_) in Eq. 7 to a parametrized synergy model:

(15) $$f_{mean}(x_{1},x_{2}|\alpha )=1/2\left(f_{2\to 1}(x_{1},x_{2}|\alpha )+f_{1\to 2}(x_{1},x_{2}|\alpha )\right),$$

which we refer to as *f*_mean_ (*x*_1_, *x*_2_ǀ α). As *f*_2→1_ (*x*_1_, *x*_2_ǀ α) and *f*_1→2_ (*x*_1_, *x*_2_ǀ α) do not fulfill the Synergy Desideratum, *f*_mean_ (*x*_1_, *x*_2_ǀ α) also does not fulfill it.

To investigate the difference between the two models *f*_2→1_ (*x*_1_, *x*_2_) (Eq. 5) and *f*_1→2_ (*x*_1_, *x*_2_) (Eq. 6) we treat compound one and two based on the difference in slopes in the conditional responses (for more detailed information on the different parameters in Hill curves, refer to **Supplementary Material**). Instead of speaking of the first and second compound, we speak of the smaller and larger one, referring to the order of steepness. Therefore, we use models Eq. 13 and Eq. 14, but categorize the compounds based on the slope parameter of their conditional response curves. This results in *f*_large→small_ (*x*_1_, *x*_2_ǀ α) and *f*_small→large_ (*x*_1_, *x*_2_ǀ α).

Analogously, we extend the Geary model *f*_geary_ (*x*_1_, *x*_2_) to a synergy model and refer to it as *f*_geary_ (*x*_1_, *x*_2_ǀ α). Based on a comment of Geary (2012), the two explicit models *f*_2→1_ (*x*_1_, *x*_2_) and *f*_1→2_ (*x*_1_, *x*_2_) yield the same surface under the LACC but do rarely in practice. Therefore, it cannot be determined whether a response that lies between the two surfaces is synergistic or antagonistic and hence should be treated as non-interactive. Thus, if α from *f*_1→2_ (*x*_1_, *x*_2_ǀ α) and α from *f*_2→1_ (*x*_1_, *x*_2_ǀ α) are of equal sign, the synergy score of that model is computed as the mean of those two parameters. In case the two synergy parameters are of opposite sign, the synergy score is set to 0:

(16) $$\alpha_{\text{geary}}=\{\begin{array}{ll} \frac{1}{2}(\alpha_{1\to 2}+\alpha_{2\to 1}) & if\;sign(\alpha_{1\to 2})=sign(\alpha_{2\to 1})\\ 0 & \text{else} \end{array}.$$

Next, to extend the null reference model following the principle of Bliss Independence, we extend Eq. 8 to

(17) $$g_{\text{bliss}}(x_{1},x_{2}|\alpha )=g_{1}(x_{1})+g_{2}(x_{2})-(1+\alpha )g_{1}(x_{1})g_{2}(x_{2}).$$

The motivation for this model is that any interaction between the two compounds is caught in the interaction term of the two conditional responses. In case of no interaction, the synergy parameter α = 0, which leads to (1 + α) = 1, and results in no deviance from the null reference model. As we use the formulation of Eq. 9 due to measuring the effect as survival, we reformulate Eq. 17 analogously as we did to get from Eq. 8 to Eq. 9: by replacing *g_i_* (*x_i_*) with 1 – *f*_i_ (*x*_i_) Hence, Eq. 17 takes the form:

(18) $$f_{bliss}(x_{1},x_{2}|\alpha )=f_{1}(x_{1})f_{2}(x_{2})+\alpha \left(1-f_{1}(x_{1})\right)\left(1-f_{2}(x_{2})\right)$$

This model does satisfy the requirement of no influence of the synergy parameter on conditional doses: *f*_bliss_ (*x*_1_, 0ǀ α) = *f*_1_ (*x*_1_) and *f*_bliss_ (0, *x*_2_ǀ α) = *f*_2_ (*x*_2_) as *f_i_* (0) = 1. In case of synergy, the interactive effect is expected to be larger, therefore, α being positive. If the compound combination has an antagonistic effect, the interaction term is expected to be negative. For extreme α, the parametric approach leads to responses outside of the range 0 ≤ *y* ≤ 1, e.g. *f*_bliss_ (*x*_1_, *x*_2_)→ − ∞ if α→−∞. The same holds for the formulations of Loewe Additivity. The implicit formulation becomes impossible to match and for the explicit formulations, the dose expression within brackets of *f*_2→1_ (*x*_1_, *x*_2_ǀ α) becomes negative. Additionally, α > 1 is not possible for *f*_CI_ (*x*_1_, *x*_2_ǀ α), as the left-hand side of Eq. 12 can not be negative. Such behavior is also known from other models, e.g. for the Greco flagship model for negative synergy scores (Greco et al., 1995, p. 365–366, and Figure 26). Hence, we will limit α to the range of −1 to 1.

Despite of the Synergy Desideratum being violated for the models that build up on the Loewe Additivity principle, there is no further effect on the model comparison presented in *Results* as conditional doses are excluded when computing the synergy score (see *Lack-of-Fit Synergy* and *Fitting the Synergy Parameter*).

#### Lack-of-Fit Synergy

The second method to measure synergy investigated here is to compute the lack-of-fit of the measured response of a combination of compounds to the response of a null reference model derived from the conditional responses. We refer to this synergy value as γ:

(19) $$\gamma =\int_{\min(x_{2}>0)}^{\max(x_{2})}\int_{\min(x_{1}>0)}^{\max(x_{1})}\left(\hat{y}(x_{1},x_{2}|\Theta )-y(x_{1},x_{2})\right)d\log(x_{1})d\log(x_{2}),$$

with *ŷ* (*x*_1_, *x*_2_ ǀ ϴ) the estimated effect with parameters ϴ of the fitted conditional responses following any non-interactive model and *y* the measured effect. Note that *ŷ* (ϴ) and *y* are dependent on the concentration combination (*x*_1_, *x*_2_). This method was used in the AstraZeneca DREAM challenge (Menden et al., 2019) with the General Isobole Equation as null reference model and can be found in (Di Veroli et al., 2016). Computing the volume has the advantage of taking the experimental design into account in contrast to simply taking the mean deviance over all measurement points, which is independent of the relative positions of the measurements. We also used a synergy value calculated from the mean deviance and it clearly performed worse (data not shown). The synergy value varies for different dose transformations. For example, the computed null-reference surface (and hence the synergy value) will be different for the same experiment if a log-transformation is applied to the doses or not.

In all, we have introduced six null reference models, five of them building up on the concept of Loewe Additivity and one on Bliss Independence. We further have introduced two methods to compute synergy, the parametric one and the lack-of-fit method, where both synergy parameters α and γ are positive if the record is synergistic, negative, if antagonistic. This results in 12 synergy model–method combinations: the parametric ones, *f*_CI_ (*x*_1_, *x*_2_ǀ α) (Eq. 12), *f*_large→small_ (*x*_1_, *x*_2_ǀα), and *f*_small→large_ (*x*_1_, *x*_2_ǀα) (Eq. 13, Eq. 14, dependent on the slope parameters) together with their mean, *f*_mean_ (*x*_1_, *x*_2_ǀ α) (Eq. 15), *f*_geary_ (*x*_1_, *x*_2_ǀ α) (computation of α_geary_ explained in Eq. 16) and *f*_bliss_ (*x*_1_, *x*_2_ǀ α) (Eq. 17). For the lack-of-fit method, we take as the null reference: *f*_GI_ (*x*_1_, *x*_2_) (Eq. 4), *f*_large→small_ (*x*_1_, *x*_2_) and *f*_small→large_ (*x*_1_, *x*_2_) (Eq. 5, Eq. 6), with the Explicit Mean Equation, *f*_mean_(*x*_1_, *x*_2_) (Eq. 7), *f*_geary_ (*x*_1_, *x*_2_) (analogously to Eq. 16) and *f*_bliss_ (*x*_1_*x*_2_) (Eq. 9).

#### Fitting the Synergy Parameter

Before applying the two methods presented in *Parametrized Synergy* and *Lack-of-Fit Synergy*, we normalize and clean the data from outliers. In a first step we normalize all records to the same value, *y*_0_, the measured response at zero dose concentration from both compounds. Second, we discard outliers using the deviation from a spline approximation. Third, we fit both conditional responses of each record, namely the responses of each compound individually, to a pair of Hill curves (Eq. S1, **Supplementary Material**). We fit the response at zero dose concentration for both Hill curves. This gives the parameter set $\Theta =\{y_{0},\;y_{\infty ,\;1},y_{\infty ,\;2},e_{1},\;e_{2},\;s_{1},\;s_{2},\}\;$ for each record. More details are given in **Supplementary Material**.

We apply the two different methods to calculate the synergy parameters α and γ to each record. First, for the parametrized synergy models, we apply a grid search for α, for α ∈[−1, 1] with a step size of 0.01, minimizing the sum of squared errors. This gives the value of α for which the squared error between the *i^th^* measured effect *y^(i)^* and *i^th^* expected effect $\hat{y}\left(x_{1}^{\left(i\right)},\;x_{2}^{\left(i\right)}\;|\alpha ,\;\Theta \right)$ is minimal:

(20) $$\underset{\alpha }{\min}\sum_{i=1,\text{with }x_{1}^{\left(i\right)}\neq 0\text{ and }x_{2}^{\left(i\right)}\neq 0}^{N}{\left(\hat{y}(x_{1}^{\left(i\right)},x_{2}^{\left(i\right)}|\alpha ,\Theta )-y^{\left(i\right)}\right)}^{2},$$

**Figure 1:**
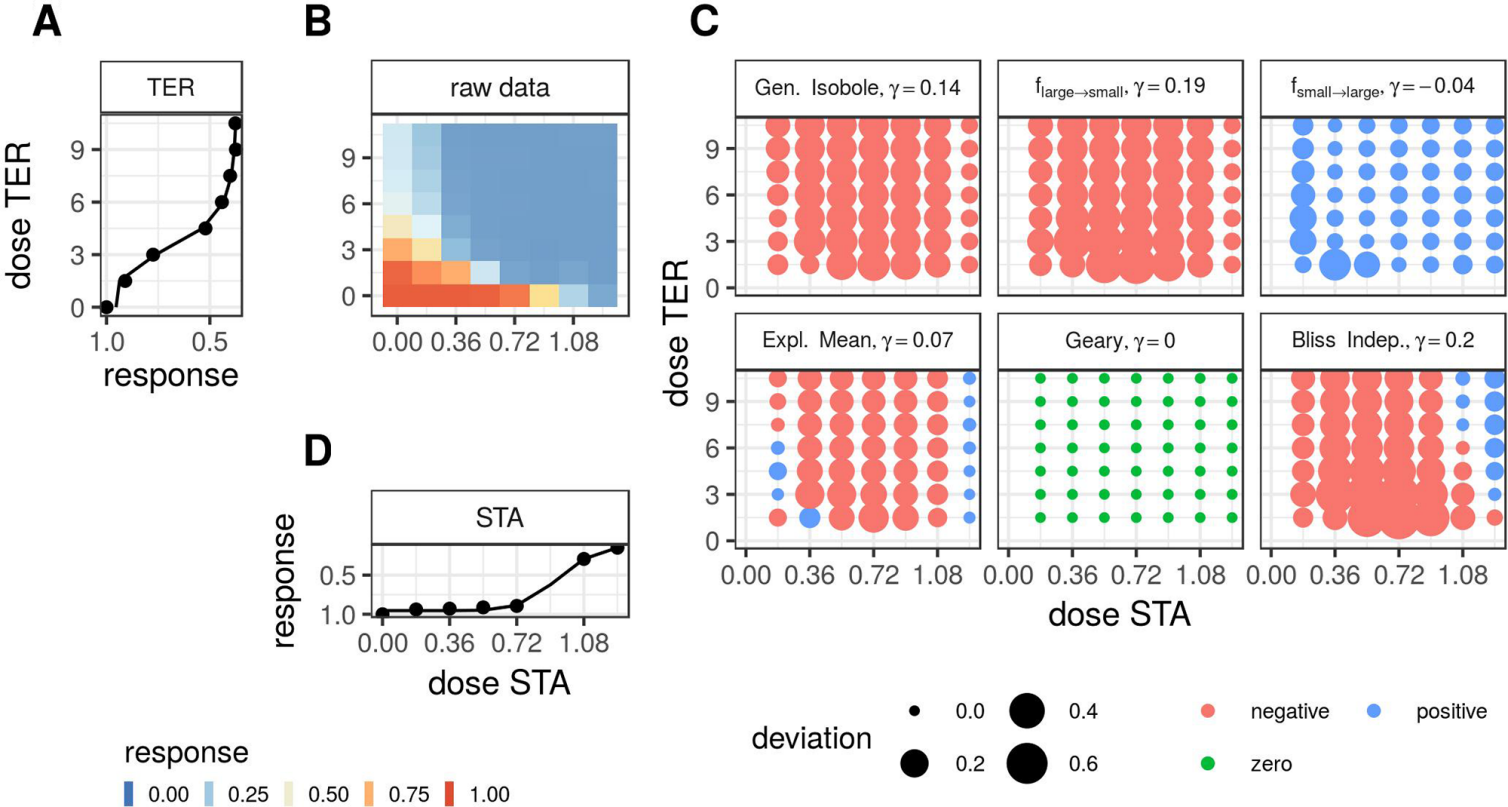
Description of the analysis steps of the lack-of-fit method for the compound pair TER and STA from the Cokol dataset. This compound pair is categorized as synergistic according to Cokol et al. (2011). The raw response data of the record is depicted in panel **(B)**. The response data normalized by the read at zero dose concentration (lower left). The degree of relative cell growth is colored from high to low values in red to blue. Step 1: compute Hill curves for conditional responses: Fit a Hill curve to the conditional responses, based on the raw reads of the single dose responses (lower and left outer edges). The fitted Hill curves are shown with the original raw data as points in panel **(A)**, which is rotated by 90 degrees, such that the vertical *x*-axis is parallel to the *y*-axis of panel **(B)**, since both axes denote the same doses of the same compound, and in panel **(D)**, which is flipped along the horizontal *x*-axis. Step 2: compute expected non-interactive response for all six models: not shown. Step 3: compute difference between measured data **(C)** and expected data from all six null reference models: shown in panel **(C)**. The direction of difference is shown by color (red for negative and blue for positive, green for zero). The larger the degree of difference, the larger the bullet, and vice versa. Step 4: compute integral *γ* over the differences: For every model, the synergy score *γ* is depicted in the title of each matrix in panel **(C)**.

Note that we exclude the conditional responses that we used to fit Θ from the minimization. Second, we apply the lack-of-fit method from Di Veroli et al. (2016), where synergy is measured in terms of the integral difference in log space of measured response and surface spanned by the non-interactive models in *Theory*, as given in Eq. 19. For the calculation of the integrals, we apply the trapezoidal rule Press et al. (1989). To compute the synergy score γ for the *f*_geary_ (*x*_1_, *x*_2_) model, we compute the integral over all data points for which the difference between expected effect or *f*_2→1_ (*x*_1_, *x*_2_) or *f*_1→2_ (*x*_1_, *x*_2_) and the measured effect are of the same sign. If they are of opposite sign, the difference is set to zero. In **Figure 1** we summarize the most important steps of the analysis for a synergistic example. In **Supplementary Material**, **Figure S5**, the same is shown for an antagonistic record.

### Material

To evaluate the two methods introduced in *Parametrized Synergy* and *Lack-of-Fit Synergy*, we apply them to two datasets of compound combination screening for which a categorization into the three synergy cases is provided.

The Mathews Griner dataset is a cancer compound synergy study by Mathews Griner et al. (2014). In a one-to-all experimental design, the compound ibrutinib was combined with 463 other compounds and administered to the cancer cell line TMD8 of which cell viability was measured. The dataset is published at https://tripod.nih.gov/matrix-client/. Each compound combination was measured for five different doses, decreasing from 125 to 2.5 µM in a four-fold dilution for each compound alongside their conditional effects, resulting in 36 different dose combinations. The categorization of this dataset comes from a study by Yadav et al. (2015), in which every record was categorized based on a visual inspection.

The Cokol dataset comes from a study about fungal cell growth of the yeast *Saccharomyces cerevisiae* (strain By4741), where Cokol et al. (2011) categorized the dataset. In this study the influence on cell growth was measured when exposed to 33 different compounds that were combined with one another based on promising combinations chosen by the authors, resulting in 200 different drug–drug–cell combinations. With an individually measured maximal effect dose for every compound, the doses administered decrease linearly in seven steps with the eight dose set to zero, resulting in an 8 × 8 factorial design.

Based on the longest arc length of an isobole that is compared to the expected longest linear isobole in a non-interactive scenario, where Loewe Additivity serves as null reference model, each record was given a score. In more detail, from the estimated surface of a record assuming no interaction, the longest contour line is measured in terms of its length and direction (convex or concave). A convex contour line leads to the categorization of a record as synergistic and the arc length of the longest contour line determines the strength of synergy. A concave contour line results in an antagonistic categorization with its extent being measured again as the length of the longest isobole. Thus the Cokol dataset not only comes with a classification but also with a synergy score similar to α or γ.

To our knowledge, these two datasets are the only high-throughput ones with a classification into the three synergy classes: antagonistic, non-interactive and synergistic. Both datasets are somewhat imbalanced because interactions are rare (Borisy et al., 2003; Zhang et al., 2007; Farha and Brown, 2010). The distribution of the classification is listed in **Table 1**. We obtained both categorizations after personal communication with the authors Yadav et al. (2015) and Cokol et al. (2011). For the purpose of comparing the synergy models, we consider these two classifications as ground truth.

**Table 1:** Number of cases categorized as synergistic, antagonistic or non-interactive in the two datasets Mathews Griner and Cokol.

	Synergistic	Antagonistic	Non-interactive
Mathews Griner	121	90	252
Cokol	50	68	82

## Results

Using the two methods of computing the synergy score, the parametric one (*Parametrized Synergy*) and the lack-of-fit one (*Lack-of-Fit Synergy*), we compute synergy scores for all records of the two datasets introduced in *Material*.

### Kendall Rank Correlation Coefficient

Having computed the synergy scores α and γ from the two different methods as described in *Fitting the Synergy Parameter*, we compute the Kendall rank correlation coefficient, which is also known as Kendall's tau coefficient and was originally proposed by Kendall (1938). This coefficient computes the rank correlation between the data as originally categorized by Yadav et al. (2015) and Cokol et al. (2011) and the computed synergy scores resulting from the two methods introduced in *Parametrized Synergy* and *Lack-of-Fit Synergy*. For the analysis, we rank synergistic records highest at rank 3, followed by non-interactive at rank 2 and antagonistic lowest at rank 1. Due to the many ties in rank, the Kendall rank correlation coefficient cannot take a value higher than 0.75 for Mathews Griner and 0.8 for Cokol, even if a perfect ranking was given. An overview of the Kendall rank correlation coefficients is given in **Tables S2** and **S3** in **Supplementary Material**.

**Figure 2:**
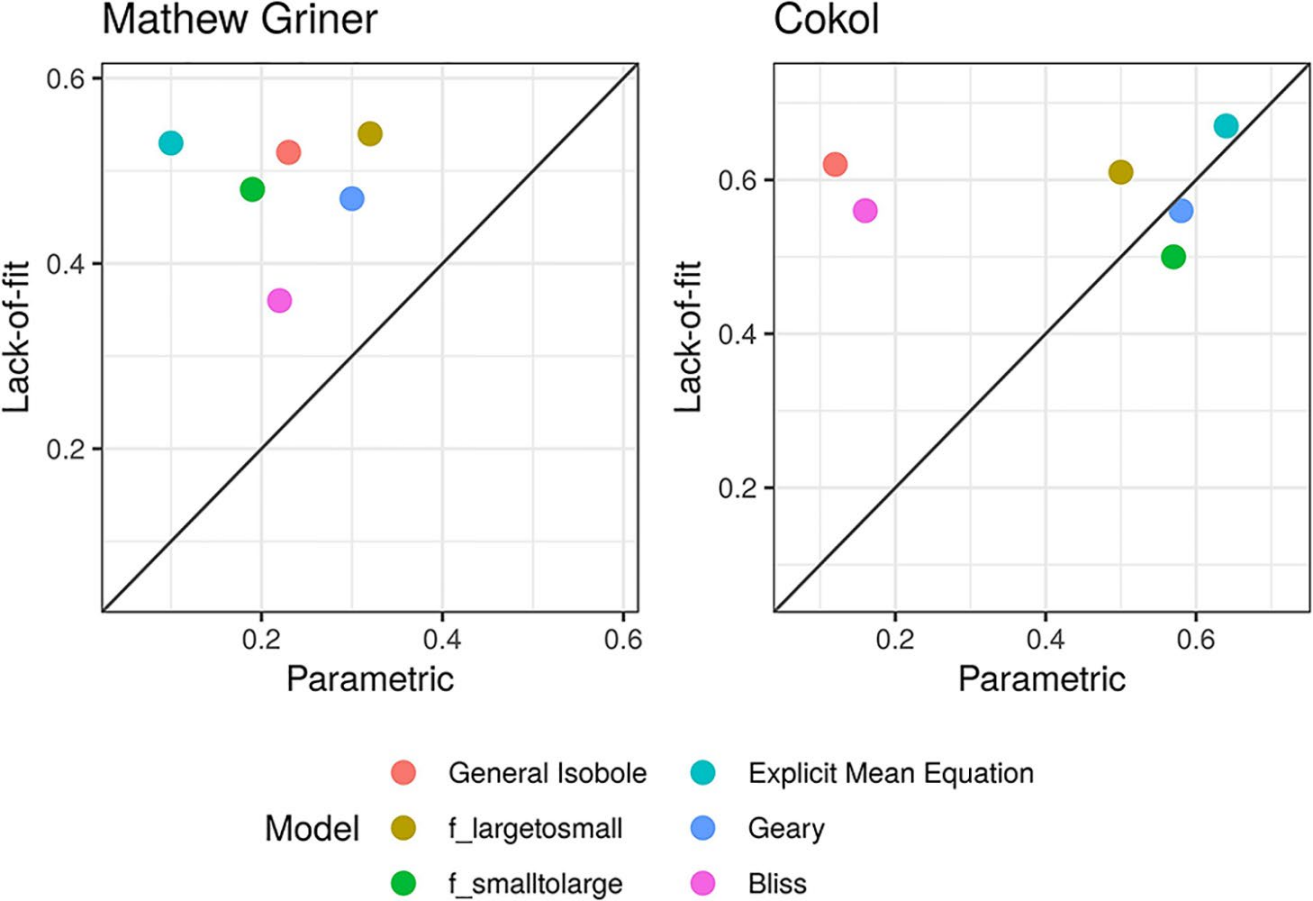
Scatter plot of Kendall rank correlation coefficient for both datasets, Mathews Griner (left) and Cokol (right). The Kendall correlation measures the rank correlation of the original categorization and the computed synergy scores. The higher the correlation, the more similar the score ranking. The correlation values from the synergy scores α, computed with the parametric approach, are plotted on the *x*-axis and those from the lack-of-fit approach are plotted on the *y*-axis. Each model is depicted in a different color. To guide the eye, the diagonal is plotted. If a data point is above the diagonal, the Kendall rank correlation coefficient from the lack-of-fit method is higher than that from the parametric method, and vice versa. Without exception, the Kendall rank correlation coefficients are all higher for the synergy scores *γ*, which are computed with the lack-of-fit method, than those based on the α scores computed with the parametric method.

To compare the parametric and lack-of-fit methods, we plot the correlation values as a scatter plot per method (see **Figure 2**) with the values from the parametric method plotted on the *x*-axis and those from the lack-of-fit method on the *y*-axis. Most of the points scatter in the upper left triangle, above the diagonal line. This shows that the lack-of-fit method outperforms the parametric method. This holds for all models applied to the Mathews Griner dataset and also for all models but *f*_geary_ (*x*_1_,*x*_2_|α) and *f*_small→large_ (*x*_1_*x*_2_|α) applied to the Cokol dataset. For both datasets, the highest correlation scores result from those null reference models that are based on the Loewe Additivity principle. The Bliss null reference model performs worst for the Mathews Griner set for both methods. For the Cokol data it is the second worst model. To a certain extent this can be explained due to the classification of the Cokol dataset being based on the isobole length relative to non-interactive isoboles, which is a Loewe Additivity type analysis. As the categorization of the Mathews Griner dataset is based on visual inspection, we cannot explain the bad performance of *f*_bliss_ (*x*_1_,*x*_2_) for that dataset. On both datasets, *f*_GI_ (*x*_1_,*x*_2_), *f*_large→small_ (*x*_1_,*x*_2_) and *f*_mean_ (*x*_1_,*x*_2_) perform best for the lack-of-fit method. For the Mathews Griner dataset, *f*_large→small_ (*x*_1_,*x*_2_) dominates marginally over the General Isobole Equation and Explicit Mean Equation model. For the Cokol dataset, the Explicit Mean Equation dominates for both methods.

### Scattering of Synergy Scores

**Figure 3:**
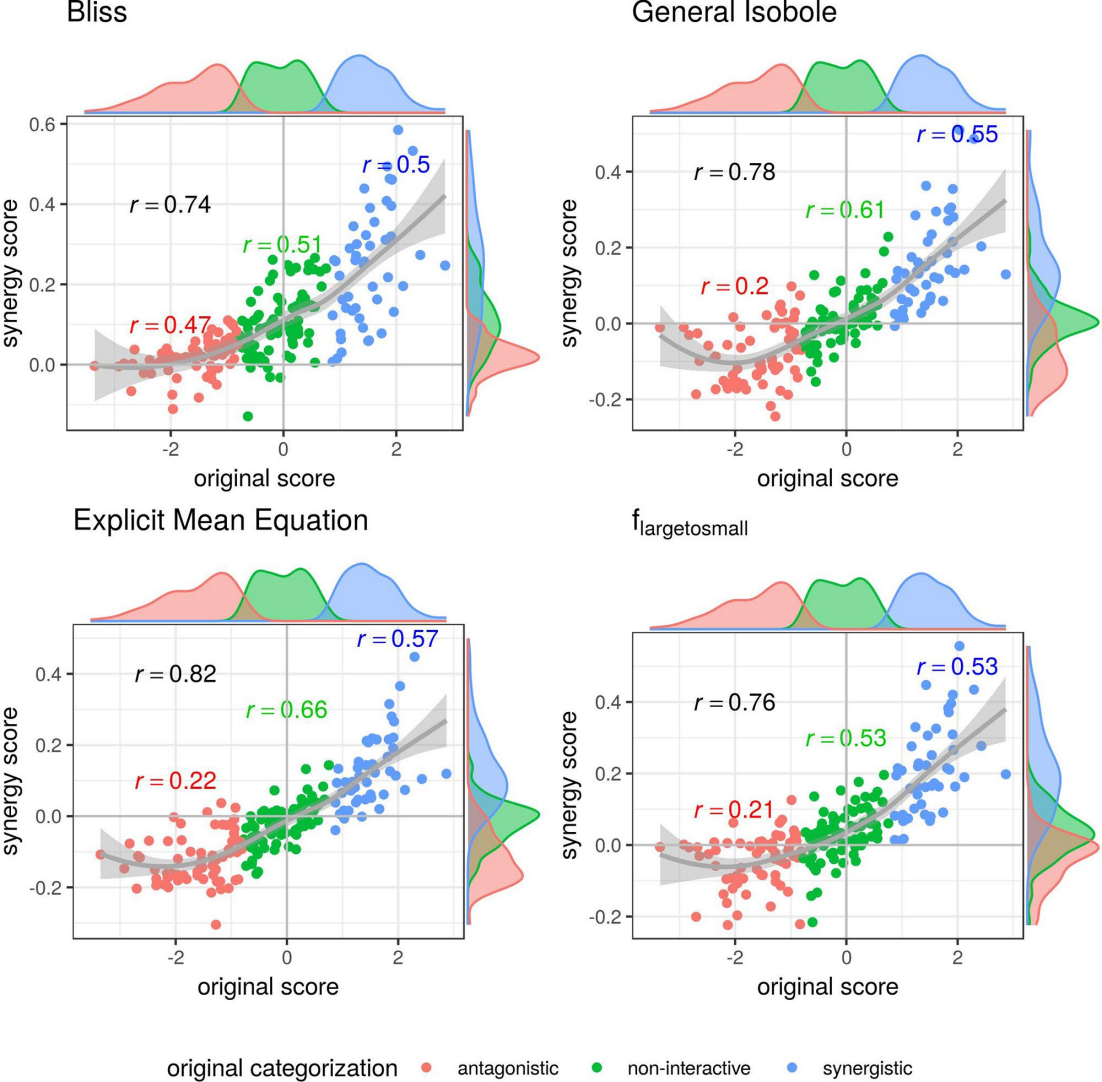
Computed synergy scores γ of the Cokol data of the best models according to the Kendall in rank correlation coefficient and the receiver operating characteristic (ROC) analysis in **Supplementary Material** in comparison to the original scores from Cokol et al. (2011). The data points are colored based on the original categorization. For all three categories, synergistic, non-interactive, and antagonistic, the Pearson correlation is depicted between the original scores in that category and the computed synergy scores in the respective color. Additionally, we depict the local polynomial regression fitting of all scores (in gray). The histograms of the scores are plotted on the axis, separated by color based on the original categorization. Synergy scores γ based on the Explicit Mean Equation model show the highest correlation with the original scores.

**Figure 4:**
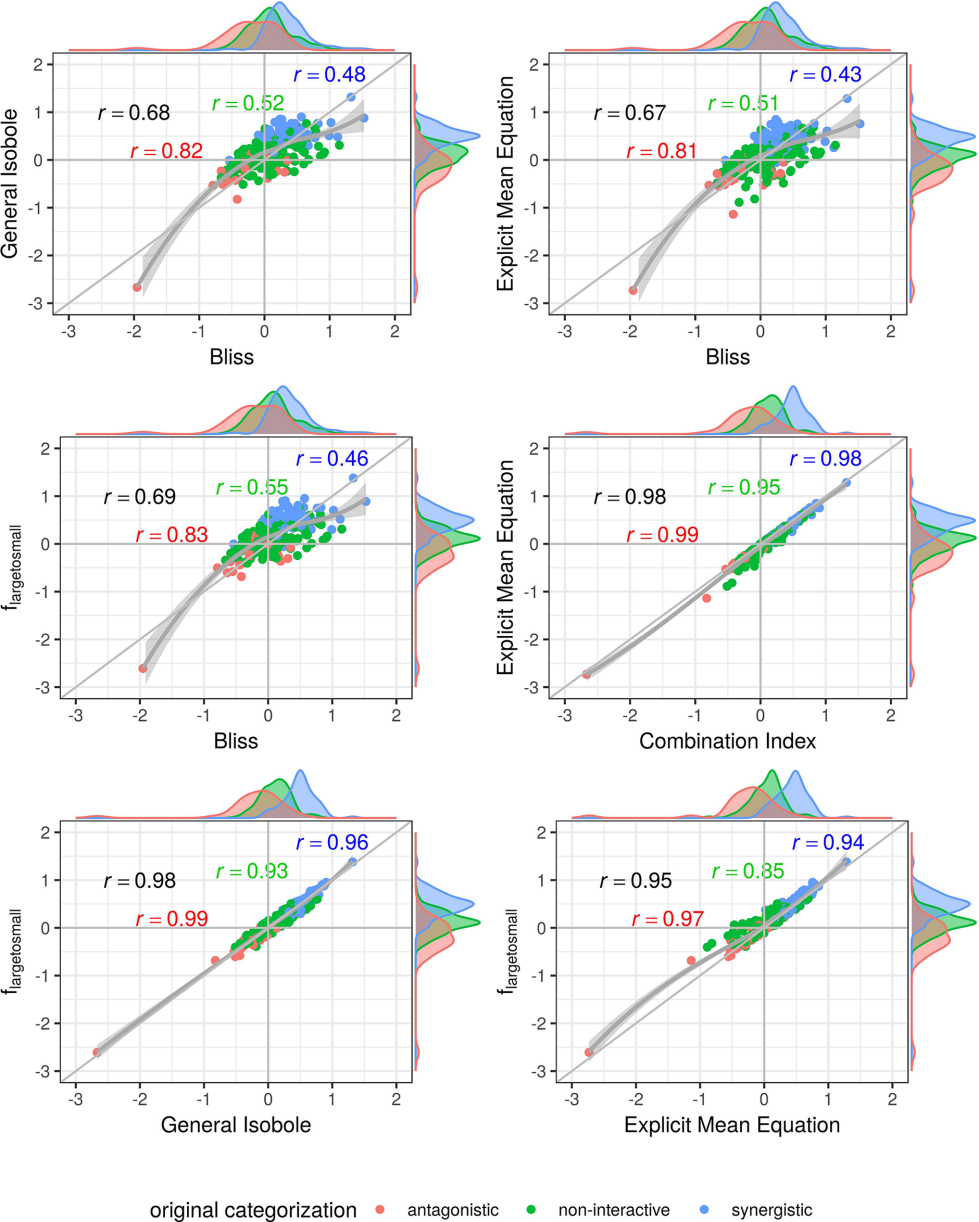
Scatter plot of synergy scores γ of the Mathews Griner dataset. The scores are computed with the lack-of-fit method. Displayed are the four best models according to the Kendall rank correlation coefficient and ROC analysis in *Kendall Rank Correlation Coefficient* and **Supplementary Material**. The scores of one model are depicted on the *x*-axis and the other on the *y*-axis. The original categorization is highlighted in different colors. The Pearson correlation score between the synergy scores are depicted by color for every categorization and the overall Pearson correlation is depicted in black. To guide the eye, the axis at 0, the diagonal and local polynomial regression fitting are depicted in grey. The histograms of the scores are plotted on the axis, separated by color based on the original categorization. The three models based on the Loewe Additivity principle show highest correlation (center right and lower row). All comparison with *f*_bliss_(*x*_1_,*x*_2_) show lowest correlation (first three cases). There is a large difference between the correlation between the additive models and the comparison of Bliss Independence by roughly 0.3.

**Figure 5:**
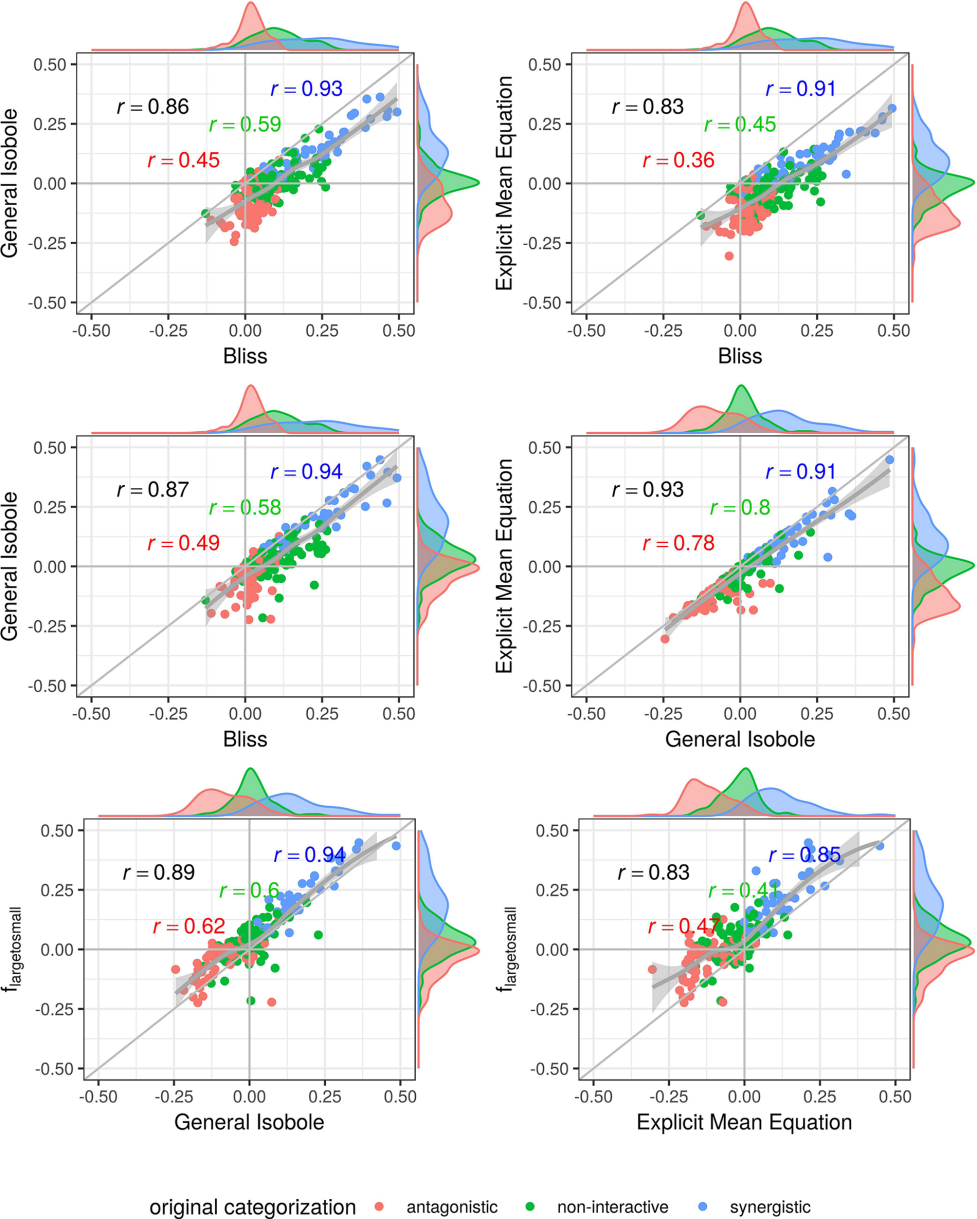
Scatter plot of synergy scores γ of the Cokol dataset. The scores are computed with the lack-of-fit method. Displayed are the four best models according to the Kendall rank correlation coefficient and ROC analysis in *Kendall Rank Correlation Coefficient* and **Supplementary Material**. The scores of one model are depicted on the *x*-axis and the other on the *y*-axis. The original categorization is given based on colour. The Pearson correlation score between the synergy scores are depicted by color for every categorization and the overall Pearson correlation is depicted in black. To guide the eye, the axis at 0, the diagonal and the local polynomial regression fitting are depicted in grey. The histograms of the scores are plotted on the axis, separated by color based on the original categorization. *f*_mean_(*x*_1_, *x*_2_) and *f*_GI_(*x*_1_, *x*_2_) show highest correlation (center right), *f*_bliss_(*x*_1_, *x*_2_) shows lowest (first three comparison cases).

To further investigate the performance of the methods and null reference models, we plot the synergy scores of the best performing models based on the Kendall rank correlation coefficient analysis (*Kendall Rank Correlation Coefficient*, and an receiver operating characteristic (ROC) analysis, which we describe in detail in **Supplementary Material**) for both datasets in **Figures 3**–**5**. In all figures, the overall correlation of the compared data is depicted together with the correlation per categorization. The coloring of the scores is based on the original categorization as antagonistic, non-interactive or synergistic as provided by Yadav et al. (2015) and Cokol et al. (2011).

In **Figure 3** the synergy scores computed with the lack-of-fit method are plotted against the original synergy scores from Cokol et al. (2011). Applying the lack-of-fit method to the Bliss Independence model (Eq. 9) results in scores which are mainly above zero (**Figure 3**, upper left). Further, it can be seen in the density plots along the *y*-axis in **Figure 3**, upper left panel, and on the *x*-axis of **Figure 4**, both panels in the first row and left panel in the middle row, that the synergy scores that are computed based on the principle of Bliss Independence cannot be easily separated by categorization, making it difficult to come up with a threshold to categorize a record into one of the three synergy categories (synergy, antagonism, non-interaction) given a synergy score.

For the other three models depicted in **Figure 3**, that are based on the principle of Loewe Additivity, the synergy scores are more clearly separated. The computed scores of the synergistic records distribute nicely above zero in the upper right corner (categorized as synergistic and computed synergy scores above zero) as well as they scatter in the lower left corner for antagonistic cases. In all those three panels in **Figure 3** we see for the non-interactive records that the computed scores of those three models are both positive and negative ranging roughly between −0.1 and 0.1 symmetrically. Barely any of the computed synergy scores for antagonistic cases are positive. Therefore, the chances of a record being antagonistic if the synergy score is above zero are quite low as well as the risk of categorizing a record as antagonistic if it is synergistic.

**Figure 6:**
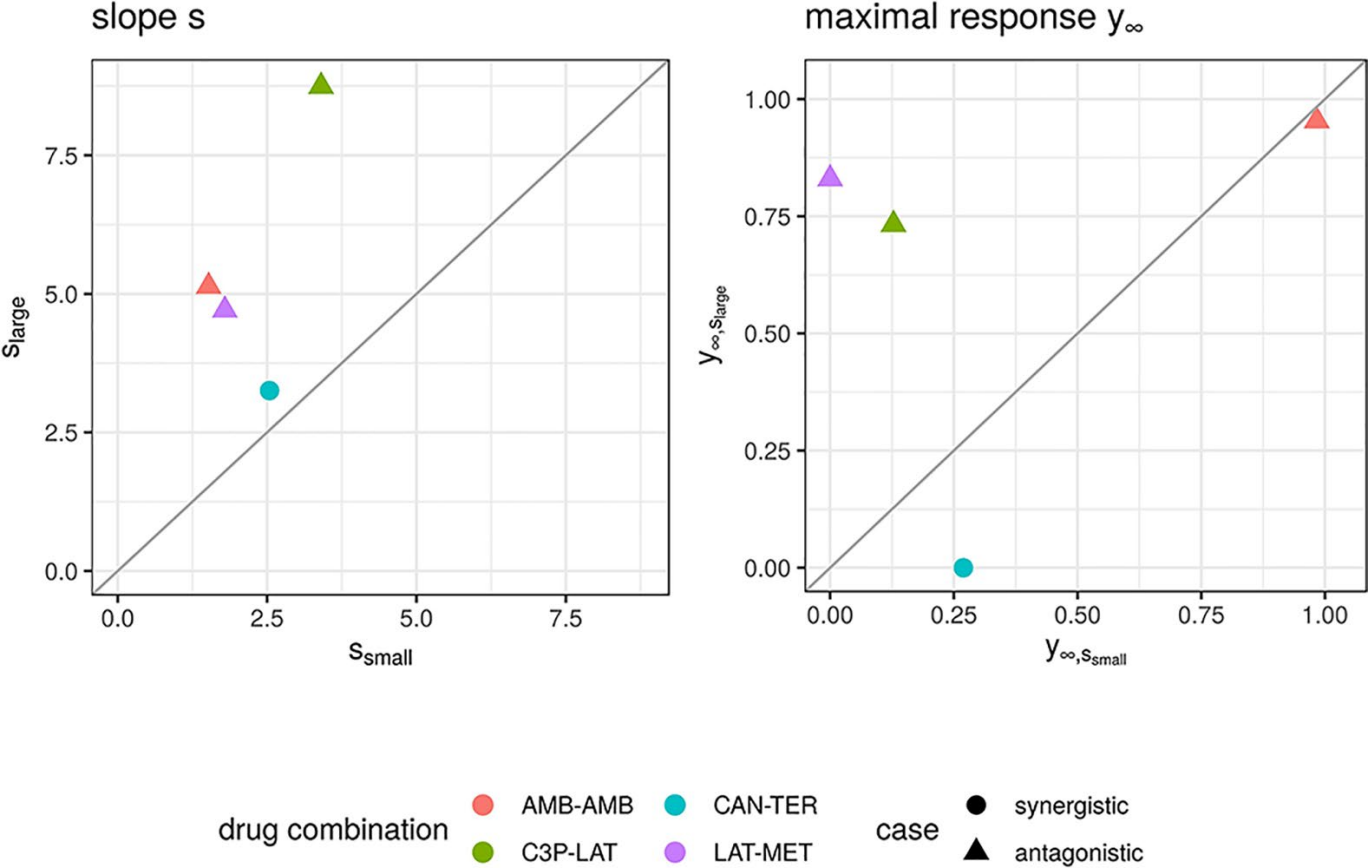
Maximal response *y*_∞_ (left) and slope parameters *s* (right) of Hill curves. Parameters are shown for the conditional responses of the four cases for which the lack-of-fit method resulted for *f*_mean_(*x*_1_, *x*_2_) and *f*_GI_(*x*_1_, *x*_2_) in a synergy score of opposite sign to its categorization from the Cokol dataset. Different records are depicted in different colours. The original categorization of each record is depicted per shape. The conditional responses of one record, and hence their Hill curve parameters, are grouped depending on size of the Hill curve parameter *s* (larger or smaller).

We further looked in detail into dose combinations for which both the *f*_GI_ (*x*_1_,*x*_2_) and *f*_mean_ (*x*_1_,*x*_2_) yield positive synergy values for antagonistic cases and into dose combinations for which the *f*_mean_ (*x*_1_,*x*_2_) model results in negative synergy values for records which are labeled as synergistic. In total we found four dose combinations. A visualization of the observed and expected responses based on the Explicit Mean Equation model is shown in **Supplementary Material**, **Figure S6**. One of them is a compound combined with itself. Hence, per definition of the Loewe Additivity, no interaction is expected. From **Figure S6**, one can see why this record was mis-categorized: for high dose combinations, a greater effect is found, which is not found for the conditional runs. Probably, the dose ranges are too small to show such effects. We looked at the conditional responses of the other three dose combinations and observed that for the originally antagonistic records (three out of four) one of the conditional responses exhibits small effects with the maximal response *y*_∞_ being above 0.65 (comp. right panel of **Figure 6**). That leads to the computed null-reference surface to be quite high and hence causes synergistic scores if any effects are measured that are smaller than [insert math here #33]. We suspect that the dose concentrations are not well-sampled and larger maximal doses should have been administered.

We looked up the three dose combinations (excluding the one where the compound is combined with itself) in the Connectivity Map (Lamb et al., 2006; Subramanian et al., 2017), which is one of the largest repositories of drug response studies. Of those, we could find three in the Connectivity Map. All of these dose combinations showed non-interactive effects on all cell lines they were tested on. The assays found in the Connectivity Map are run on cancer cell lines. The dose combinations investigated here are run on yeast. Hence, a full comparison cannot be made, but results are certainly suggestive that the compound combinations are non-interactive.

In **Figures 4** and **5**, the computed scores from different null reference models are plotted against each other. We compare the implicit formulation (General Isobole Equation) to the Bliss Independence model and the two best performing models that are based on the explicit formulation of Loewe Additivity, *f*_mean_ (*x*_1_,*x*_2_) and *f*_large→small_ (*x*_1_,*x*_2_). The coloring of the scores is based on the original categorization as antagonistic, non-interactive or synergistic as provided by Yadav et al. (2015) and Cokol et al. (2011).

In **Figure 4** the scores from the Mathews Griner dataset are plotted. In the two panels in the upper row and the left panel in the middle row Bliss Independence is compared to the other three null models that build up on the principles of Loewe Additivity. It is obvious, that the scores based on Bliss Independence are larger than those of Loewe Additivity and mainly above zero. This is due to the more conservative null reference surface as derived from Bliss Independence [see Sinzger et al. (2019), **Figure 6**]. The scores from models that are based on Loewe Additivity are very similar to each other, as they scatter along the diagonal (panels in middle right and lower row). It is difficult, though, to tell apart whether a record is synergistic or antagonistic, as non-interactive records scatter largely between −0.5 and 0.5. Only records with a computed score outside that range can be categorized as interactive. For the Cokol dataset, which serves as basis for **Figure 5**, the scores can be better separated. Despite the scores being generally smaller than those from the Mathews Griner data, the records can be easier separated, when using a Loewe Additivity based model. Additionally, we see here the similarity between these additive models given their strong correlation (right panels in middle row and both panels in lower row). Further, the scores based on *f*_large→small_ (*x*_1_,*x*_2_) achieve higher values than those from the other two Loewe Additivity based models. This becomes obvious when comparing the null-reference surfaces of those three models, as depicted in Lederer et al. (2018b). The surface spanned by *f*_large→small_ (*x*_1_,*x*_2_) spans a surface above those surfaces spanned by Explicit Mean Equation or General Isobole Equation. Therefore, in synergistic cases where the measured effect is greater, and hence the response in cell survival smaller, the difference from the null-reference surface to *f*_large→small_ (*x*_1_,*x*_2_) is greater than to the other two models. We suspect the synergy models from the Cokol dataset to be better separable due to the experimental design of the dataset. All compounds were applied up to their known maximal effect dose. This was not the case for the Mathews Griner dataset, where all compounds were applied at the same fixed dose range.

All in all, the lack-of-fit method performs better for any model when applied to the Mathews Griner dataset and mostly better for the Cokol dataset, with the exception of the *f*_small→large_ (*x*_1_,*x*_2_) and Geary model. We suggest, that the lack-of-fit should be preferred over the parametric method, due to the overall performance on both datasets. When using the lack-of-fit method, the Explicit Mean Equation model performs either second best (Mathews Griner dataset), or best (Cokol dataset). The other two well performing models, the explicit *f*_large→small_ (*x*_1_,*x*_2_) or the original implicit formulation of Loewe Additivity, the General Isobole Equation, do not perform equally well on both datasets. To exclude any bias from these models for different datasets, the Explicit Mean Equation should be preferred.

## Discussion

The rise of high-throughput methods in recent years allows for massive screening of compound combinations. With the increase of data, there is an urge to develop methods that allow for reliable filtering of promising combinations. Additionally, the recent success of a synergy study of *in vivo* mice by Grüner et al. (2016) underlines the fast development of possibilities to generate biological data. Therefore, it is all the more important to develop methods that are sound and easily applicable to high-throughput data.

In this study we use two datasets of compound combinations that come with a categorization into synergistic, non-interactive or antagonistic for each record.

Based on the fitted conditional responses, we compute the synergy scores of all records. We compare six models that build on the principles of Loewe Additivity and Bliss Independence. Those six models are used with two different methods to compute a synergy score for each record. The first method is a parametric approach and is motivated by the Combination Index introduced by Berenbaum (1977). The second method quantifies the lack-of-fit, i.e. the difference in volume between the expected response assuming no interaction and the measured response and is motivated by Di Veroli et al. (2016).

We compare the computed synergy scores from both methods, each applied with the six reference models, based on Kendall rank correlation coefficients. Based on these correlation coefficients we investigate the reconstruction of ranking of the records (see *Kendall Rank Correlation Coefficient*). We further conduct an ROC analysis (results shown in **Supplementary Material**). With this, we quantify the methods' and models' capacity to distinguish records from different categories, given a computed synergy score. Both, the Kendall rank correlation coefficient and the ROC analysis, show a superiority of those models that are based on Loewe Additivity relative to those based on Bliss Independence. From those additive models the Explicit Mean Equation is the overall best performing model for both datasets.

For the above comparison of the six null reference models and the two methods, we rely on the underlying categorization of both datasets. All performance metrics are based on how well the predicted synergy scores agree with the underlying categorization. The categorizations of both datasets were created very differently from one another. On one hand, the Mathews Griner dataset was categorized on a visual inspection, on account of which we cannot be certain about the assumptions made that guided the decision making process. On the other hand, the categorization of the Cokol dataset is based on the principle of Loewe Additivity. This leads to the natural preference of null models that are based on Loewe Additivity over those based on Bliss Independence, which we find back in our analysis. Irrespective of the origin of the classification, we stress that the labels were provided to us by independent researchers and hence were not biased in any way to favor the Explicit Mean Equation model.

Note that we conduct the research only on combinations of two compounds. Research on higher-order combinations is usually performed with the principle of Bliss Independence (Zimmer et al., 2016; Katzir et al., 2019) as its extension is straight-forward. The General Isobole Equation is also easily extensible to more than two compounds (theoretical work in Gennings and Carter, 1995 and an analysis of three combinations can be found in Fang et al., 2017; Tosi et al., 2018). An extension of the explicit formulations of Loewe Additivity to more than two compounds would increase the number of explicit equations (Eq. 5 and Eq. 6) equal to the number of compounds used. Hence, all six null-reference models can be extended to higher-order compound combinations. The same holds for the parametric and lack-of-fit method. It has to be kept in mind, that the number of drug combinations grows exponentially with the number of drugs. A full experimental design with complete set of dose combinations is, to the best of our knowledge, only reported in the work of Fang et al. (2017) and Tosi et al. (2018).

Meanwhile, it is shown in Russ and Kishony (2018) that Bliss Independence maintains accuracy when increasing the number of compounds that are combined with each other. Loewe Additivity, however, loses its predictive power for an increasing number of compounds.

The comparison of the parametric method with the lack-of-fit method shows a superiority of the lack-of-fit method. To recall, the motivation behind the parametric approach was the statistical advantages of such an approach. It allows to define an interval around α = 0 in which a compound combination can be considered additive. For the lack-of-fit method, such statistical evaluation can not be done directly, but could be performed on the basis of bootstrapping.

Chou and Talalay (1977) measure the interaction effect locally for a fixed ratio of doses of both compounds that are supposed to reach the same effect, say one unit of the first compound causes the same effect as two units of the second compound, which results in the dose combination of 1:2. Along this fixed ratio of doses, they compute the left-hand side of Eq. 3 given the two doses *x*_1_ and *x*_2_ that are assumed to reach a fixed effect y* together with $x_{i}^{*}$ being the dose of compound *i* that reaches the fixed effect alone. For the fixed dose ratio, they run over all expected effects, usually from zero to one. A geometric interpretation of that method is depicted in (Greco et al., 1995, Figure 7, p. 341). The resulting values of the left-hand side of Eq. 3 are analyzed graphically: all computed values are plotted versus the expected fixed effect y* = [0,1]. Values higher than one exhibit synergistic behavior, values below one antagonism. This method allows for results that show antagonistic behavior for, say, smaller effects, as well as synergistic behavior for higher effects, or vice versa. That such a behavior of switching from antagonistic behavior in one region to synergistic behavior in another can occur was also shown in Norberg and Wahlström (1988). With one synergy score, as used throughout this paper, we do not provide such a measure for local antagonism and synergism. Our main motivation in this study is to provide a single synergy score that allows for fast filtering of interesting candidates for more in-depth research. To extend that idea, the standard deviation could be taken into account, as in a *t*-value or Z-score. Additionally, the superior lack-of-fit method is much faster and simpler to implement than the parametric one.

Finally, to asses how distinguishable the synergy scores γ are, we visualize the synergy scores based on the underlying category (*Scattering of Synergy Scores*). The synergy scores from the lack-of-fit method can, based on their sign, reliably be categorized as synergistic or antagonistic. For records categorized as non-interactive, the computed synergy scores are positive as well as negative. For the two datasets, we saw different extents of separation between those γ-scores, which makes it difficult to generalize the results. All in all, the differentiation from no interaction poses a more difficult task as choosing the threshold is arbitrary.

During the analysis, we observed higher synergy scores when applying the Bliss Independence principle as null reference model. This is due to the more conservative null reference surface as derived from Bliss Independence [see exemplary comparison of isoboles from most of the models discussed here in (Sinzger et al., 2019, **Figure 6**)]. Due to the synergy scores being relatively high, a differentiation between categories based on the synergy score poses a bigger challenge. There is a strong overlap of synergy scores from all three categories. Additionally, most of the synergy scores γ, that are computed with the lack-of-fit method, are above zero. Different ranges of synergy scores for both datasets make it additionally difficult to assess synergy or antagonism for a record based on the unique information of the synergy score.

We want to emphasize the performance benefit of the recently introduced Explicit Mean Equation (Lederer et al., 2018b) over the implicit formulation in form of the General Isobole Equation. On both datasets, it is the overall best performing model when compared to the provided categorizations. The explicit formulation of this additive model was shown to speed up computation by a factor of 250 [see Lederer et al., 2018b, **Figure S1**)]. Together with the implementation of the lack-of-fit method, which is easier to implement and a lot faster than the parametric method, this combination of model improvement and method can be of great benefit for the research community.

Although the performance of models and methods are consistent across the two (quite different) datasets considered in this study, reliable comparison of different models and methods would benefit from the availability of drug screening datasets that are available with ground truth labeling.

## Data Availability Statement

The datasets analyzed in this manuscript are not publicly available. Requests to access the datasets should be directed to Bhagwan Yadav, ghagwan.yadav@helsinki.fi (https://tripod.nih.gov/matrix-client/); Murat Cokol, cokol@sabanciuniv.edu.

## Author Contributions

TD conceived the research. SL performed the data analysis. All authors wrote or contributed to the writing of the manuscript.

## Funding

This work was supported by the Radboud University and CogIMon H2020 ICT-644727. We acknowledge institutional funding from the Max Planck Society.

## Conflict of Interest

The authors declare that the research was conducted in the absence of any commercial or financial relationships that could be construed as a potential conflict of interest.
