# Supplementary material for: Additive Dose Response Models: Defining Synergy
Source: Front Pharmacol. 2019 Nov 26;10:1384. doi: 10.3389/fphar.2019.01384 (PMC6901947; doi:10.3389/fphar.2019.01384)
Supplement: Supplementary file 1 [file DataSheet_1.pdf]

Supplementary Material:
Additive Dose Response Models: Defining Synergy

Simone Lederer*, Tjeerd H.M. Dijkstra and Tom Heskes
*Correspondence:
Author Name: Simone Lederer
slederer@cs.ru.nl

1 CONDITIONAL DOSE RESPONSE CURVES

A common approach for modeling monotonic dose-response curves f_j with $j \in \{1,2\}$ is the Hill curve (Hill, 1910), also referred to as the sigmoid function. The Hill model is, due to its good fit to many sources of data, the most widely applied model for fitting compound responses (Goutelle et al., 2008). It has a sigmoidal shape with little change for small doses but with a rapid decline in response once a certain threshold is met. For even larger doses the effect asymptotes to a constant maximal effect. Two exemplary Hill curves are depicted in Fig. S1. There are several parameterizations of the Hill curve. We use the following throughout

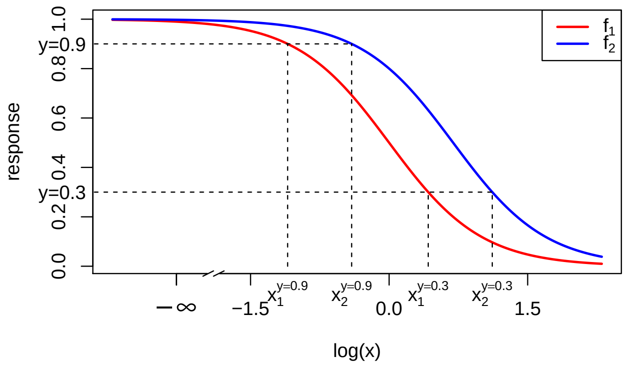

Figure S1. Dose-response curves (red and blue) as Hill curves (Eq. S1). For the exemplary responses of 0.3 and 0.9 the different doses x_1 and x_2 reaching that effect are shown (dashed lines). The dose-response curves differ only in EC_{50} with $e_1 = 2$ and $e_2 = 1$. Values of the other parameters are $y_0 = 1$, $y_\infty = 0$ and $s = 2$. To highlight the sigmoidal shape of a Hill curve in log-space, the logarithmic concentration space is depicted.

this study to fit conditional responses:

$$f(x) = y_\infty + \frac{y_0 - y_\infty}{1 + (\frac{x}{e})^s}, \tag{S1}$$

where y_0 is the response at zero dose and y_∞ the maximal response of the cells to the compound, e the dose concentration reaching half of the maximal response and s the steepness of the curve. Eq. S1 is equivalent

to the parametrization used in the drc package (Ritz and Strebig, 2016), the so-called four parameter log-logistic model. By our definition of the Hill curve, a positive s leads to a descending Hill curve.

2 DATA CLEANING, FITTING OF HILL CURVE AND PARAMETER ESTIMATION FOR IMPLICIT MODELS

First, we normalize all records by the measured response at zero dose concentration from both compounds, y_0. Second, we conduct an outlier analysis of the normalized responses by fitting a spline surface and deleting outliers to discard them. Third, we then fit the conditional responses of the cleaned data to Hill curves.

We fit a general additive model (GAM) to the normalized raw data using thin plate splines (Wood, 2006), not transforming the doses in any way. The surfaces of those fitted thin plate splines span the checkerboards of every record and data points with too large absolute residual values are rejected. For fitting the splines we use method gam() of the mgcv-package (Wood, 2011), defining the smooth terms within the gam formulae with the method s(). We set the dimension of the basis, that is used to represent the smooth term to $k = 30$ fixed knots.

The threshold to reject data points is at five times the inter-quantile range of all residuals of a given record. Every data point with an absolute residual above that threshold is discarded. For the Mathews Griner data, this leads to 18 records out of the 466 (less than 4%) where a mean of 1.28 outliers were excluded per record with an overall of 23 data points excluded, which is less than one percent of the overall data. A maximum of 6 outliers was detected once. Similarly, we excluded on average 2.48 data points for the Cokol data on 52 of the total 200 ($\leq 25\%$) records with a maximum of 13 data points and an overall of 129 data points excluded, which is about 1% of all data points.

To fit the two conditional responses of a record to two Hill functions of the form of Eq. S1 we use the drc package (Ritz et al., 2019). Unlike other synergy analyses such as (Yadav et al., 2015), the response at zero concentration y_0 is not fixed to 1 but merely constrained to be the same for both response curves. The other Hill parameters, y_∞, s and e are fitted for both compounds individually. In case the asymptote parameter y_∞ is below zero for any of the two Hill curves, the conditional response of that compound is refitted to a two-parameter model with y_∞ set to zero and y_0 kept from the fitting of both compounds together. This is the case for 43 records of the Mathews Griner dataset and 125 records of the Cokol dataset. We exclude records for which any of the Hill curve parameters slope or EC50 are negative ($s < 0, e < 0$). This is the case for 187 records for the Mathews Griner dataset (133 records with negative slope s, 88 records with negative EC50 value e, out of which there are 34 records with negative slope and negative EC50 value), which is roughly 40% of all records. More details follow below.

The $f_{\text{GI}}\left(x_1, x_2\right)$ model is an implicit model for the response y. Therefore, a root finder is used to find a response $\hat{y}^{(i)}$ given concentrations $\left(x_1^{(i)}, x_2^{(i)}\right)$ and parameters describing the Hill curves of the conditional responses, $\Theta = \{y_0, y_{\infty,j}, e_j, s_j\}$, We used the standard implementation of a root finder in the R stats package, uniroot() (R Core Team, 2016), which is based on the Brent-Dekker-van Wijngaarden algorithm (Press et al., 1989, Chapter 9). As convergence criterion we used 1.22×10^{-4} within a maximum of 1000 iterations.

2.1 Sensitivity of model performance to inter-quantile range

In a previous version of this article, we cleaned the data to three times the inter-quantile range instead of five. With this smaller inter-quantile range we removed in the Mathews Griner dataset in total 199 data points instead of 23, and in the Cokol dataset 623 instead of 129. The performance of the Mathews Griner dataset for the lack-of-fit method slightly decreased, whereas the overall performance for the Cokol dataset increased.

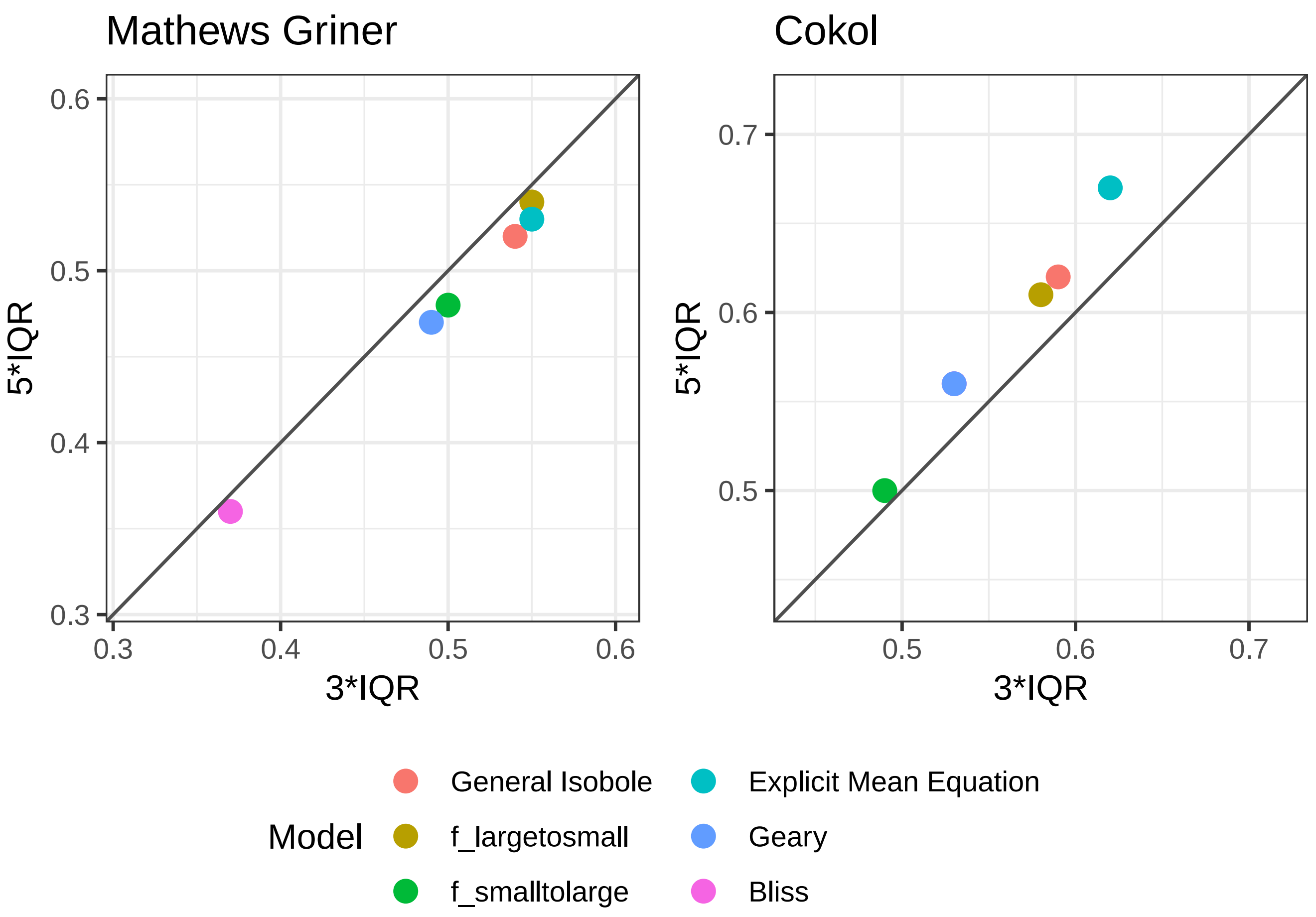

Figure S2. Scatter plot of Kendall rank correlation coefficient for both datasets, Mathews Griner (left) and Cokol (right), comparing the performance of the lack-of-fit method for different data-cleaning thresholds. The Kendall rank correlation coefficient values resulting from the cleaned data with three times the inter-quantile range are plotted on the x-axis and those from the data cleaned with a threshold of five times inter-quantile range are plotted on the y-axis. Each model is depicted in a different colour. To guide the eye, the diagonal is plotted.

As a note, approximately the same number of records were excluded for the analysis due to two issues: i) negative slopes of at least one of the conditional dose response curves, or ii) the root-finder for the $f_{\text{CI}}(x_1, x_2|\alpha)$ model not converging (no convergence after 1000 iterations). These issues are independent from data cleaning with three or five times the inter-quantile range (see Appendix 4, Table S12 - Table S15).

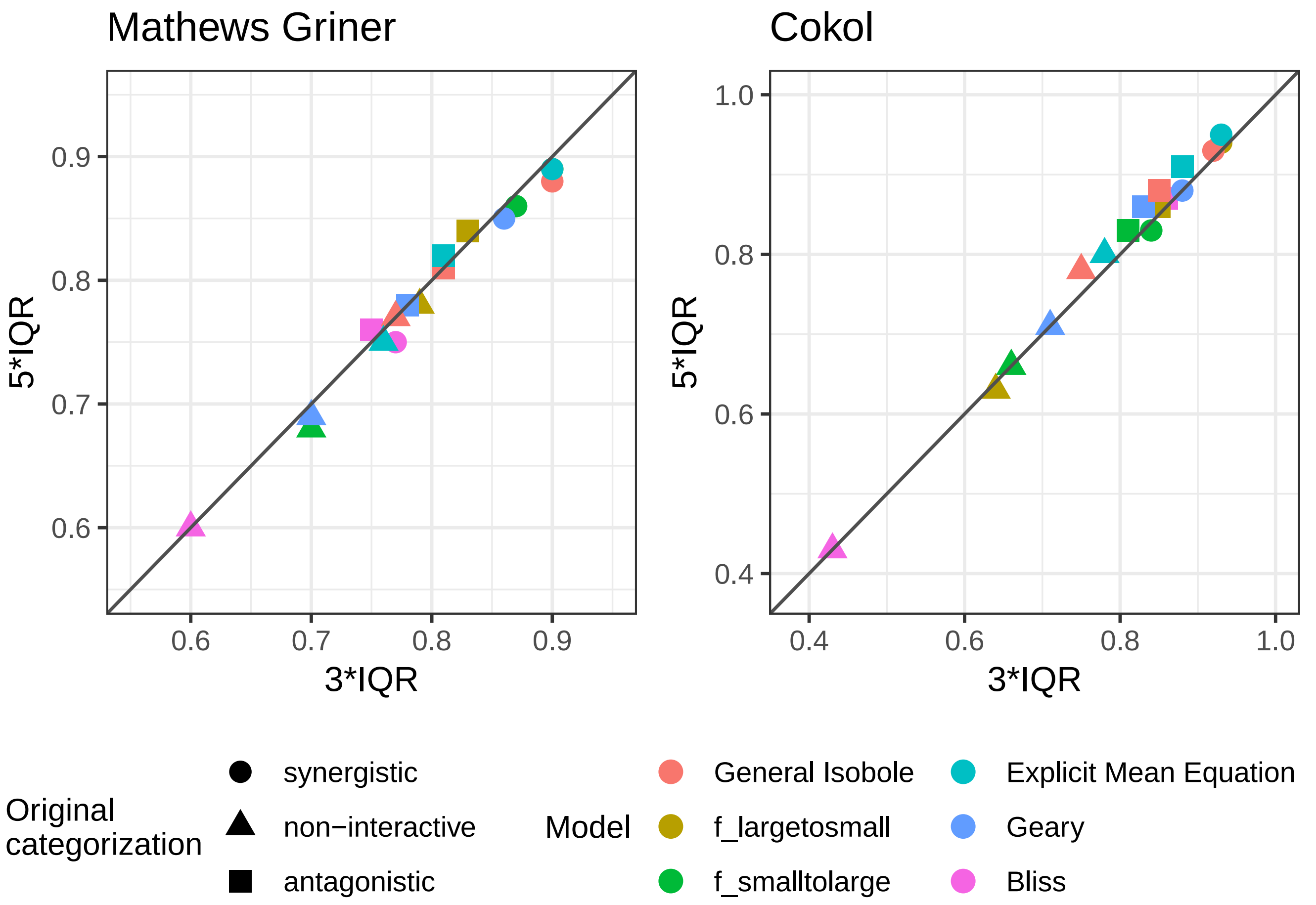

Figure S3. Scatter plot of ROC analysis for both datasets, Mathews Griner (left) and Cokol (right), comparing the performance of the lack-of-fit method for different data-cleaning thresholds. The AUC values resulting from the cleaned data with three times the inter-quantile range are plotted on the x-axis and those from the data cleaned with a five times inter-quantile range are plotted on the y-axis. AUC values from different models are shown in different colors. AUC values comparing the different categories are depicted in different shapes, where the naming of the shape represents the category that is compared to the remaining two. To guide the eye, the diagonal is plotted. The more a datapoint is above the diagonal, the better the performance of the data cleaned with a threshold of five times the inter-quantile range, and vice versa.

2.2 Handling records with negative slope or EC50 values

Roughly 40% (187) of the records of the Mathews Griner dataset were excluded in the study because of a negative slope or EC50 parameter. This is due to a suboptimal choice of doses. We observed two types of sub-optimality: first, the maximal dose can be too small to induce a significant change in response. Due to the noise in measurements, negative slope and EC50 parameters are fitted. This is the case for 34 records. A second type of sub-optimality is observed when the maximal effect is already reached for the second dose (the first dose is always zero). This is the case for the remaining 153 records.

Although we could not fit two reasonable Hill curves to these records, we can still use both methods, lack-of-fit and parametric, to quantify synergy. They both only require two mathematically well-defined conditional response curves. Here, we define a conditional response for cases with negative slope or negative EC50 parameter according to Table S1.

s	e	$y(x)$
< 0	< 0	$y_0 \quad \forall \quad x$
< 0	> 0	y_∞ for $x \neq 0$
> 0	< 0	y_∞ for $x \neq 0$

Table S1. Response curves for cases where the Hill model fit leads to negative slope or negative EC50 values.

With the above definition for conditional response curves, we investigated the 187 previously excluded records in detail. We computed the lack-of-fit synergy values γ for those 187 records and for the entire dataset of 466 records. For two of these records, the $f_{\mathrm{GI}}\,(x_1, x_2)$ model did not converge. The Kendall rank correlation coefficient values are given in Appendix 4, Table S16. The inclusion of those datasets results in lower Kendall rank correlation coefficients relative to the original analysis. The coefficients decrease by roughly 0.05 when averaged over all models.

3 ROC-ANALYSIS

In high-throughput synergy studies, one generally screens for promising candidates that exhibit a synergistic or antagonistic effect. Those promising candidates are then investigated in more detail with genetic assays and other techniques. To determine how well the underlying null reference models result in distinguishable synergy scores, we conduct an ROC analysis (receiver operating characteristic), comparing the estimated synergy scores with the class categorization that is given for both datasets. A standard ROC analysis applies to binary classification, where cases are compared to controls. In this study, we have three classes: synergistic, antagonistic and non-interactive. We therefore compare each class to the combination of the other two, e.g. synergistic as cases versus the antagonistic and non-interactive combined as control. Typically, in ROC analyses, the cases rank higher than the controls. When treating the class antagonistic as case compared to the control synergistic and non-interactive we change all signs of the synergy scores. Therefore, the ranking of synergy scores is reversed and antagonistic synergy scores rank higher. Problems arise when comparing non-interactive cases to the control synergistic and antagonistic as their values should lie between the two control classes. Therefore, the absolute value of the estimated synergy scores is taken, which allows a ranking where the synergy scores of the non-interactive records should rank lower than the other synergy scores. Additionally, we can again multiply all synergy scores with minus one to revert the order of scores such that the cases rank higher.

The AUC values (area under the curve) are reported in Table S4 - Table S7 in Appendix 4. For completeness, and based on the critique of Saito and Rehmsmeier (2015) to use PRC-AUC (precision/recall area under the curve) values for imbalanced datasets, the PRC-AUC values are also computed and can be found in Table S8 - Table S11 in Appendix 4.

Analogously to the previous section, we depict the AUC values for both datasets in scatter plots (Fig. S4) with AUC values based on the parametric approach depicted on the x-axis and those based on the lack-of-fit approach on the y-axis. The underlying null reference models are shown by color. The different comparisons, such as synergistic versus non-interactive and antagonistic, are depicted by shape of the plot symbol.

From Fig. S4, the dominance of the lack-of-fit approach over the parametric one is as apparent for the Mathews Griner dataset as from Fig. 2. With regard to the comparison of the different cases, visualized in shape, the AUC values from the comparison of the synergistic cases to the non-interactive and antagonistic

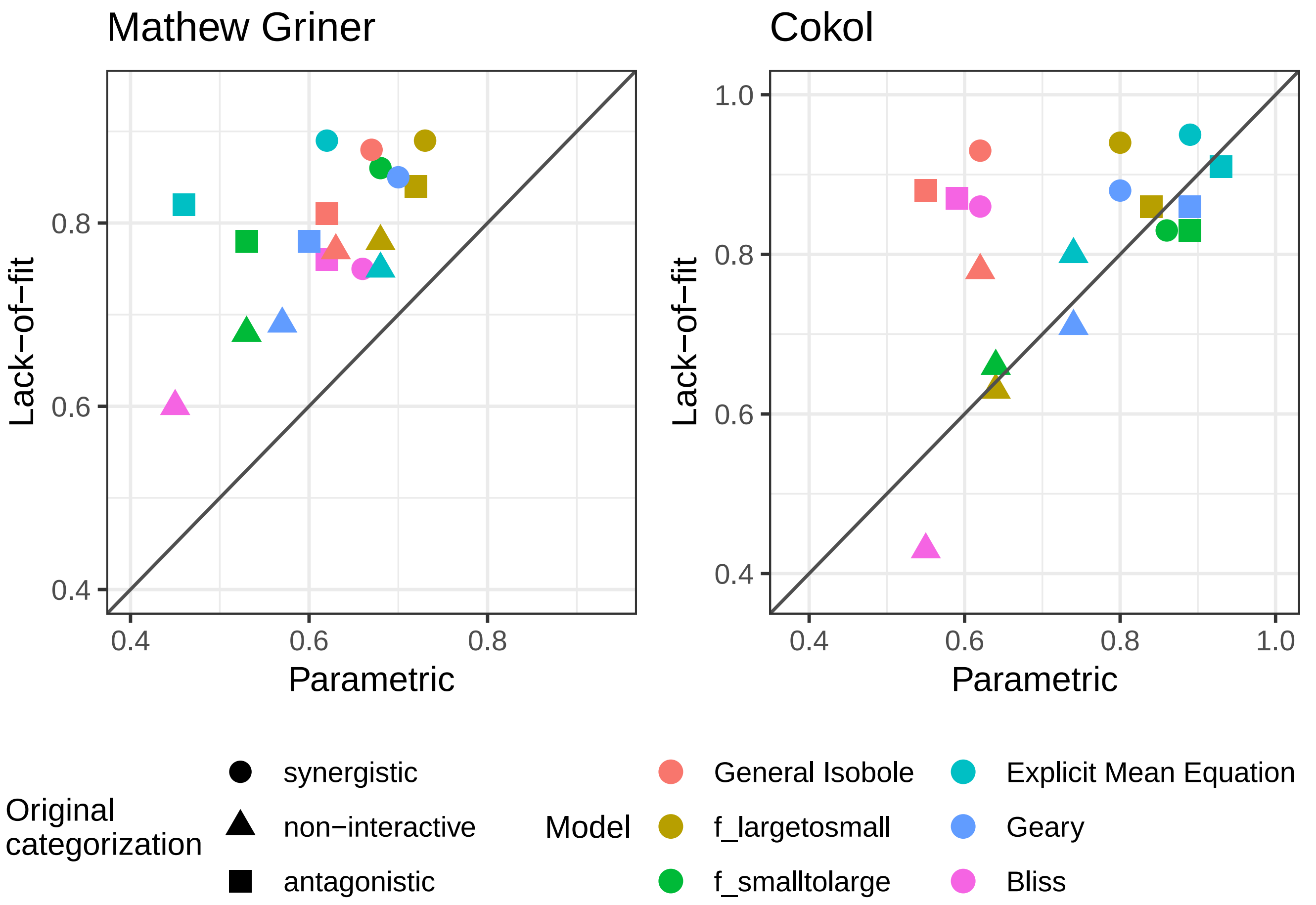

Figure S4. Scatter plot of ROC values for both datasets, Mathews Griner (left) and Cokol (right). The ROC values resulting from the parametric approach are plotted on the x-axis and those from the lack-of-fit approache are plotted on the y-axis. Each model is depicted in a different color. The three different comparisons, of one case versus the remaining two, are depicted in different shapes. To guide the eye, the diagonal is plotted. If a data point is above the diagonal, the ROC value from the lack-of-fit method is higher than that from the parametric method, and vice versa. Except for the non-interactive comparison of the Bliss Independence model, the synergy scores γ from the lack-of-fit method always result in higher ROC values than those computed based on the synergy scores α from the parametric method.

controls, score the highest values around 0.9. The comparison of the non-interactive cases to the interactive ones score the lowest.

As the overall highest AUC scores result from the lack-of-fit method, we have a closer look at those for both datasets (Table S5 and Table S7 in Appendix 4). For the antagonistic case, the values range around 0.80 for the Mathews Griner dataset and around 0.85 for the Cokol dataset. AUC values of the non-interactive case range around 0.75 for both datasets. The AUC values for the synergistic case for both datasets range around a value of 0.90 with one outlier of 0.75 for the Bliss Independence model on the Mathews Griner dataset.

Overall, the lack-of-fit outperforms the parametric method on the Mathews Griner dataset. For the lack-of-fit method, both the $f_{\text{large}\to\text{small}}\left(x_1, x_2\right)$ and Explicit Mean Equation perform best on the Mathews Griner dataset for synergistic cases, and a clear dominance of $f_{\text{large}\to\text{small}}\left(x_1, x_2\right)$ over the Explicit Mean

Equation for antagonistic and non-interactive cases. On the second dataset, the Cokol dataset, Explicit Mean Equation performs overall best for both methods.

We attribute the differences in performances of methods and models on the two datasets to the differences in the experimental design for these datasets. For the Cokol dataset, all compounds were applied up to their maximal effect dose. In the Mathews Griner dataset, all compounds were applied with the same fixed dose range.

REFERENCES

Cokol, M., Chua, H. N., Tasan, M., Mutlu, B., Weinstein, Z. B., Suzuki, Y., et al. (2011). Systematic exploration of synergistic drug pairs. *Molecular Systems Biology* 7, 544. doi:10.1038/msb.2011.71

Goutelle, S., Maurin, M., Rougier, F., Barbaut, X., Bourguignon, L., Ducher, M., et al. (2008). The Hill equation: A review of its capabilities in pharmacological modelling. *Fundamental and Clinical Pharmacology* 22, 633–648. doi:10.1111/j.1472-8206.2008.00633.x

Hill, A. V. (1910). The possible effects of the aggregation of the molecule of hemoglobin on its dissociation curves. *Journal of Physiology* 40, iv–vii

Press, W. H., Teukolsky, S. A., Vetterling, W. T., and Flannery, B. P. (1989). *Numerical Recipes: The Art of Scientific Computing*, vol. 31 (Cambridge: Cambridge University Press), 3 edn. doi:10.1137/1031025

R Core Team (2016). R: A Language and Environment for Statistical Computing

Ritz, C., Jensen, S. M., Gerhard, D., and Streibig, J. C. (2019). *Dose-Response Analysis Using R*, vol. 10 (Boca Raton, Florida : CRC Press, [2019]: Chapman and Hall/CRC). doi:10.1201/b21966

Ritz, C. and Strebig, J. C. (2016). drc: Analysis of Dose-Response Curves

Saito, T. and Rehmsmeier, M. (2015). The precision-recall plot is more informative than the ROC plot when evaluating binary classifiers on imbalanced datasets. *PLoS ONE* 10, 1–21. doi:10.1371/journal.pone.0118432

Wood, S. N. (2006). *Generalized additive models: an introduction with R* (Boca Raton: Chapman and Hall/CRC), 2 edn.

Wood, S. N. (2011). Fast stable restricted maximum likelihood and marginal likelihood estimation of semiparametric generalized linear models. *Journal of the Royal Statistical Society: Series B (Statistical Methodology)* 73, 3–36. doi:10.1111/j.1467-9868.2010.00749.x

Yadav, B., Wennerberg, K., Aittokallio, T., and Tang, J. (2015). Searching for Drug Synergy in Complex DoseResponse Landscapes Using an Interaction Potency Model. *Computational and Structural Biotechnology Journal* 13, 504–513. doi:10.1016/j.csbj.2015.09.001

4 SUPPLEMENTARY TABLES

model	lack-of-fit	parametric
$f_{\text{GI}}(x_1, x_2)$	0.52	0.23
$f_{\text{large}\rightarrow\text{small}}(x_1, x_2)$	**0.54**	**0.32**
$f_{\text{small}\rightarrow\text{large}}(x_1, x_2)$	0.48	0.19
$f_{\text{mean}}(x_1, x_2)$	0.53	0.10
$f_{\text{geary}}(x_1, x_2)$	0.47	0.30
$f_{\text{bliss}}(x_1, x_2)$	0.36	0.22

Table S2. Kendall rank correlation coefficient of Mathews Griner data set.

model	lack-of-fit	parametric
$f_{\text{GI}}(x_1, x_2)$	0.62	0.12
$f_{\text{large}\rightarrow\text{small}}(x_1, x_2)$	0.61	0.50
$f_{\text{small}\rightarrow\text{large}}(x_1, x_2)$	0.50	0.57
$f_{\text{mean}}(x_1, x_2)$	**0.67**	**0.64**
$f_{\text{geary}}(x_1, x_2)$	0.56	0.58
$f_{\text{bliss}}(x_1, x_2)$	0.56	0.16

Table S3. Kendall rank correlation coefficient of Cokol data set.

	synergistic	non-interactive	antagonistic
$f_{\text{GI}}(x_1, x_2)$	0.67	0.63	0.62
$f_{\text{large}\rightarrow\text{small}}(x_1, x_2)$	**0.73**	**0.68**	**0.72**
$f_{\text{small}\rightarrow\text{large}}(x_1, x_2)$	0.68	0.53	0.53
$f_{\text{mean}}(x_1, x_2)$	0.62	**0.68**	0.46
$f_{\text{geary}}(x_1, x_2)$	0.70	0.57	0.60
$f_{\text{bliss}}(x_1, x_2)$	0.66	0.45	0.62

Table S4. AUC analysis of parametric method applied to Mathews Griner dataset.

	synergistic	non-interactive	antagonistic
$f_{\text{GI}}(x_1, x_2)$	0.88	0.77	0.81
$f_{\text{large}\rightarrow\text{small}}(x_1, x_2)$	**0.89**	**0.78**	**0.84**
$f_{\text{small}\rightarrow\text{large}}(x_1, x_2)$	0.86	0.68	0.78
$f_{\text{mean}}(x_1, x_2)$	**0.89**	0.75	0.82
$f_{\text{geary}}(x_1, x_2)$	0.85	0.69	0.78
$f_{\text{bliss}}(x_1, x_2)$	0.75	0.60	0.76

Table S5. AUC analysis on lack-of-fit method applied to Mathews Griner dataset.

	synergistic	non-interactive	antagonistic
$f_{\text{GI}}(x_1, x_2)$	0.62	0.62	0.55
$f_{\text{large}\to\text{small}}(x_1, x_2)$	0.80	0.64	0.84
$f_{\text{small}\to\text{large}}(x_1, x_2)$	0.86	0.64	0.89
$f_{\text{mean}}(x_1, x_2)$	**0.89**	**0.74**	**0.93**
$f_{\text{geary}}(x_1, x_2)$	0.80	**0.74**	0.89
$f_{\text{bliss}}(x_1, x_2)$	0.62	0.55	0.59

Table S6. AUC analysis on parametric method applied to Cokol dataset.

	synergistic	non-interactive	antagonistic
$f_{\text{GI}}(x_1, x_2)$	0.93	0.78	0.88
$f_{\text{large}\to\text{small}}(x_1, x_2)$	0.94	0.63	0.86
$f_{\text{small}\to\text{large}}(x_1, x_2)$	0.83	0.66	0.83
$f_{\text{mean}}(x_1, x_2)$	**0.95**	**0.80**	**0.91**
$f_{\text{geary}}(x_1, x_2)$	0.88	0.71	0.86
$f_{\text{bliss}}(x_1, x_2)$	0.86	0.43	0.87

Table S7. AUC analysis on lack-of-fit method applied to Cokol dataset.

	synergistic	non-interactive	antagonistic
$f_{\text{GI}}(x_1, x_2)$	0.39	0.69	**0.35**
$f_{\text{large}\to\text{small}}(x_1, x_2)$	**0.62**	0.73	0.32
$f_{\text{small}\to\text{large}}(x_1, x_2)$	0.52	0.58	0.13
$f_{\text{mean}}(x_1, x_2)$	0.56	**0.74**	0.18
$f_{\text{geary}}(x_1, x_2)$	0.61	0.61	0.15
$f_{\text{bliss}}(x_1, x_2)$	0.39	0.55	0.17

Table S8. PRC-AUC analysis on parametric method applied to Mathews Griner dataset.

	synergistic	non-interactive	antagonistic
$f_{\text{GI}}(x_1, x_2)$	0.78	0.75	0.48
$f_{\text{large}\to\text{small}}(x_1, x_2)$	**0.80**	0.74	**0.55**
$f_{\text{small}\to\text{large}}(x_1, x_2)$	0.72	0.69	0.33
$f_{\text{mean}}(x_1, x_2)$	0.78	**0.76**	0.42
$f_{\text{geary}}(x_1, x_2)$	0.71	0.70	0.35
$f_{\text{bliss}}(x_1, x_2)$	0.52	0.64	0.39

Table S9. PRC-AUC analysis on lack-of-fit method applied to Mathews Griner dataset.

	synergistic	non-interactive	antagonistic
$f_{\text{GI}}(x_1, x_2)$	0.30	0.57	0.51
$f_{\text{large}\to\text{small}}(x_1, x_2)$	0.56	0.56	0.65
$f_{\text{small}\to\text{large}}(x_1, x_2)$	0.62	0.52	0.83
$f_{\text{mean}}(x_1, x_2)$	**0.71**	0.55	**0.89**
$f_{\text{geary}}(x_1, x_2)$	0.60	**0.58**	0.82
$f_{\text{bliss}}(x_1, x_2)$	0.30	0.45	0.50

Table S10. PRC-AUC analysis on parametric method applied to Cokol dataset.

	synergistic	non-interactive	antagonistic
$f_{\text{GI}}\left(x_1, x_2\right)$	0.84	0.65	0.83
$f_{\text{large}\rightarrow\text{small}}\left(x_1, x_2\right)$	**0.87**	0.46	0.72
$f_{\text{small}\rightarrow\text{large}}\left(x_1, x_2\right)$	0.63	0.56	0.72
$f_{\text{mean}}\left(x_1, x_2\right)$	**0.87**	**0.66**	**0.86**
$f_{\text{geary}}\left(x_1, x_2\right)$	0.75	0.60	0.77
$f_{\text{bliss}}\left(x_1, x_2\right)$	0.76	0.35	0.70

Table S11. PRC-AUC analysis on lack-of-fit method applied to Cokol dataset.

	synergistic	antagonistic	non-interactive	total
parametric	19	15	48	82
lack-of-fit	34	59	93	186
both	19	16	49	84

Table S12. # of excluded records from parametric and lack-of-fit method applied to the Mathews Griner dataset with a threshold of three times the inter-quantile range.

	synergistic	antagonistic	non-interactive	total
parametric	21	16	49	86
lack-of-fit	36	58	91	185
both	21	16	49	86

Table S13. # of excluded records from the parametric and lack-of-fit method applied to the Mathews Griner dataset with cleaned data of a threshold of five times the inter-quantile range.

	synergistic	antagonistic	non-interactive	total
parametric	6	4	6	16
lack-of-fit	7	5	7	19
both	6	4	6	16

Table S14. # of excluded records from parametric and lack-of-fit method applied to the Cokol dataset with cleaned data of a threshold of three times the inter-quantile range.

	synergistic	antagonistic	non-interactive	total
parametric	3	2	4	9
lack-of-fit	3	3	4	10
both	3	2	4	9

Table S15. # of excluded records from parametric and lack-of-fit method applied to Cokol dataset with cleaned data of a threshold of five times the inter-quantile range.

model	subset of 185 records	entire set of 464 records	original analysis on 279 records
$f_{\text{GI}}\left(x_1, x_2\right)$	0.37	**0.47**	0.52
$f_{\text{large}\rightarrow\text{small}}\left(x_1, x_2\right)$	0.34	0.43	**0.54**
$f_{\text{small}\rightarrow\text{large}}\left(x_1, x_2\right)$	**0.42**	0.46	0.48
$f_{\text{mean}}\left(x_1, x_2\right)$	0.37	0.43	0.53
$f_{\text{geary}}\left(x_1, x_2\right)$	0.35	0.39	0.47
$f_{\text{bliss}}\left(x_1, x_2\right)$	0.27	0.34	0.36

Table S16. Kendall rank correlation coefficients with recomputed conditional response curves according to Table S1 on 185 records with negative slope or EC50 value (left) and on entire dataset with 464 records (right).

5 SUPPLEMENTARY FIGURES

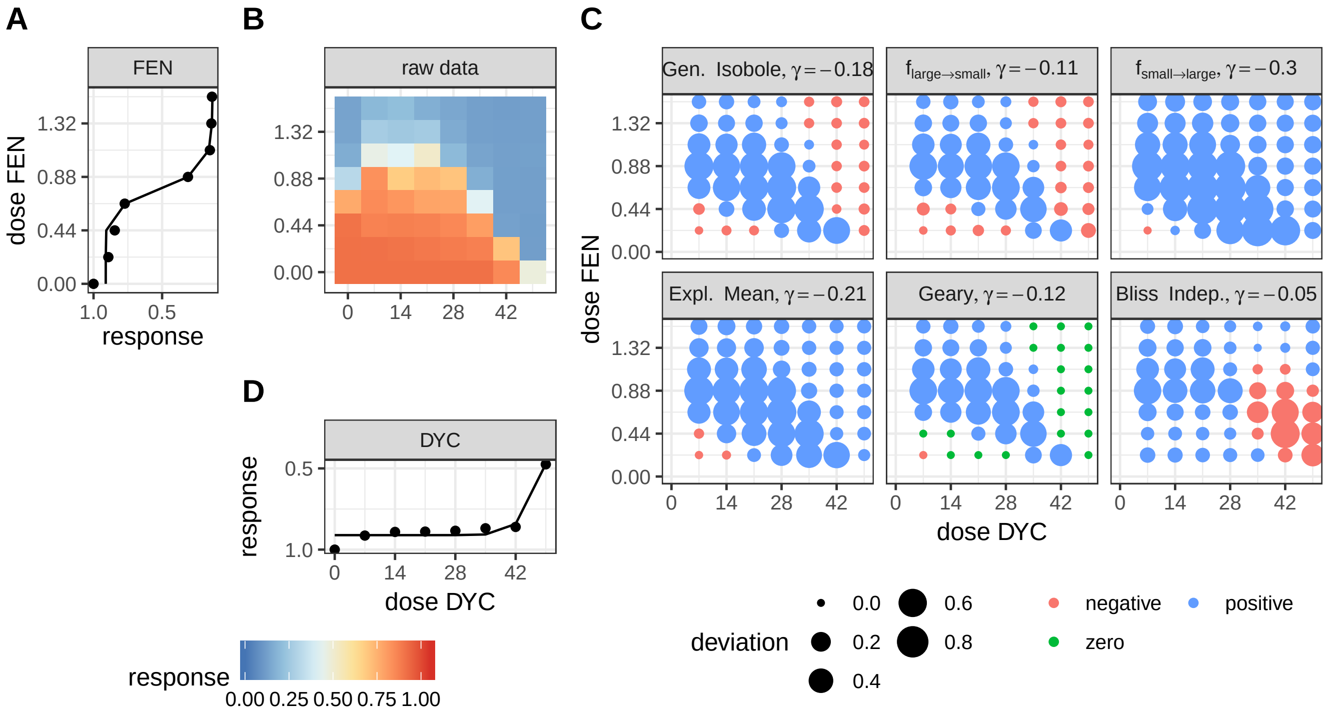

Figure S5. Description of the analysis steps of the lack-of-fit method for the compound pair FEN and DYC from the Cokol dataset. This compound pair is categorized as antagonistic according to (Cokol et al., 2011). The raw response data of the record is depicted in (B). The response data normalized by the read at zero dose concentration (lower left). In (B) the degree of relative cell growth is colored from high to low values in red to blue.
Step 1: compute Hill curves for conditional responses: Based on the raw reads of the single dose responses (lower and left outer edges) fit a Hill curve to the conditional responses. The fitted Hill curves shown in (A) and (D) with original raw data shown as points.
Step 2: compute expected non-interactive response for all six models: not shown.
Step 3: compute difference between measured data (C) and expected data from all six null reference models: shown in (C). The direction of difference is shown by color (red for negative and blue for positive, green for zero). The larger the degree of difference, the larger the bullet, and vice versa.
Step 4: compute integral γ over the differences: Over all those bullets, we then compute the integral, which gives the synergy score γ. For every model, the synergy score γ is depicted in the title of each matrix in (C).

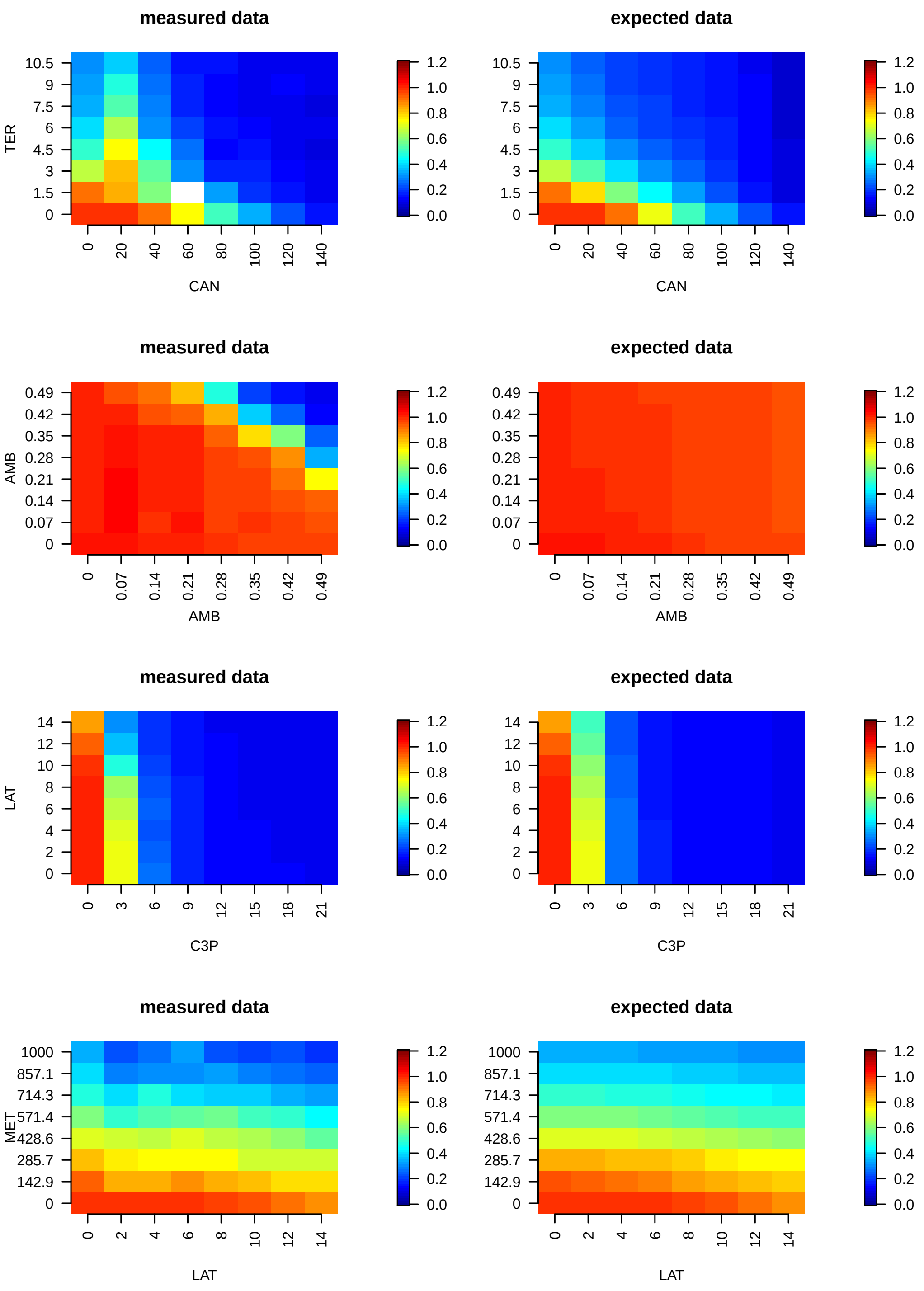

Figure S6. Raw responses (left) and expected responses from the Explicit Mean Equation model (right) of the four records from the Cokol dataset, for which the General Isobole Equation and Explicit Mean Equation gave synergy scores of opposite sign to the orignal categorization. More details on some parameters of the Hill curves can be found in Fig. 6.
